# Loss of Bardet-Biedl syndrome proteins causes synaptic aberrations in principal neurons

**DOI:** 10.1371/journal.pbio.3000414

**Published:** 2019-09-03

**Authors:** Naila Haq, Christoph Schmidt-Hieber, Fernando J. Sialana, Lorenza Ciani, Janosch P. Heller, Michelle Stewart, Liz Bentley, Sara Wells, Richard J. Rodenburg, Patrick M. Nolan, Elizabeth Forsythe, Michael C. Wu, Gert Lubec, P. Salinas, Michael Häusser, Philip L. Beales, Sofia Christou-Savina

**Affiliations:** 1 Great Ormond Street Institute of Child Health, University College London, London, United Kingdom; 2 Wolfson Institute for Biomedical Research and Department of Neuroscience, Physiology and Pharmacology, University College London, London, United Kingdom; 3 Department of Pharmaceutical Chemistry, University of Vienna, Vienna, Austria; 4 Department of Cell and Developmental Biology, University College London, London, United Kingdom; 5 Institute of Neurology, University College London, London, United Kingdom; 6 MRC Harwell Institute, Mary Lyon Centre, Harwell Campus, Oxfordshire, United Kingdom; 7 Radboud Center for Mitochondrial Medicine, Translational Metabolic Laboratory, Department of Pediatrics, Radboud University Medical Centre, Nijmegen, the Netherlands; 8 Neurodigitech, LLC, San Diego, California, United States of America; 9 Programme in Proteomics, Paracelsus Private Medical University, Salzburg, Austria; Indiana University Purdue University at Indianapolis, UNITED STATES

## Abstract

Bardet-Biedl syndrome (BBS), a ciliopathy, is a rare genetic condition characterised by retinal degeneration, obesity, kidney failure, and cognitive impairment. In spite of progress made in our general understanding of BBS aetiology, the molecular and cellular mechanisms underlying cognitive impairment in BBS remain elusive. Here, we report that the loss of BBS proteins causes synaptic dysfunction in principal neurons, providing a possible explanation for the cognitive impairment phenotype observed in BBS patients. Using synaptosomal proteomics and immunocytochemistry, we demonstrate the presence of Bbs proteins in the postsynaptic density (PSD) of hippocampal neurons. Loss of Bbs results in a significant reduction of dendritic spines in principal neurons of *Bbs* mouse models. Furthermore, we show that spine deficiency correlates with events that destabilise spine architecture, such as impaired spine membrane receptor signalling, known to be involved in the maintenance of dendritic spines. Our findings suggest a role for BBS proteins in dendritic spine homeostasis that may be linked to the cognitive phenotype observed in BBS.

## Abstract 2

In Bardet-Biedl syndrome (BBS), mutations that affect the function of primary cilia cause retinal degeneration, obesity, kidney failure, and cognitive impairment. The cognitive impairment has yet to be explained, but this study shows that loss of BBS proteins causes synaptic aberrations in principal neurons and can be partially rescued by aerobic exercise.

## Introduction

Dendritic spines are small protrusions that cover the dendrites of most principal neurons in the vertebrate central nervous system (CNS), where they typically serve as the postsynaptic part of excitatory synapses [1]. Recent studies have revealed that alterations in dendritic spines are associated with a wide range of conditions associated with cognitive impairments, ranging from rare monogenic neurodevelopmental syndromes to common psychiatric diseases, including schizophrenia and bipolar disorder [2–4]. Dendritic spine shape, size, and number are regulated in a spatiotemporal manner that is tightly coordinated with synaptic function and plasticity [5,6]. The formation, maintenance, stability and pruning of spines are firmly controlled by a wide range of surface receptors that, when activated by extracellular ligands, trigger diverse downstream signalling pathways. Membrane receptors, such as insulin-like growth factor (IGF-1R), ephrin B (EphB), and tyrosine receptor kinase B (TrkB), have profound effects on neuroplasticity in the CNS [7,8]. There are myriad ways in which the activation of these receptors may mediate neuroplasticity, including modulation of the protein kinase B (Akt)/ mammalian target of rapamycin (mTOR) pathway, macroautophagy, small GTPases activity, and glutamate receptors (GluRs) membrane expression [9].

BBS proteins are a group of ciliary proteins that, when mutated, cause a rare genetic disorder, Bardet-Biedl syndrome (BBS). BBS is a genetically heterogeneous, autosomal recessive disorder characterised by early-onset retinal degeneration, obesity, polydactyly, renal dysfunction, and cognitive impairment [10,11]. BBS was one of the first multisystem disorders ascribed to dysfunctional nonmotile cilia, microtubule-based membranous projections protruding from the cell surface of most mammalian cells, including neurons [12]. It was shown that eight highly conserved BBS proteins form a coat-like complex (‘BBSome’) that is responsible for the trafficking of signalling receptors into and out of the cilia [13]. Loss of BBS proteins affects the entry and exit of signalling receptors such as somatostatin receptor type 3 (Sstr3), melanin-concentrating hormone receptor 1 (Mchr1), dopamine receptors (D1), G-protein coupled receptor 161 (Gpr161), and brain-derived neurotrophic factor (BDNF) receptors (TrkB) [14,15]. Several reports have recently demonstrated that in addition to their ciliary function, BBS proteins play an essential role in actin cytoskeleton rearrangements, transcriptional regulation, and endosomal trafficking [16–18].

An area of growing interest is the molecular role of BBS proteins in cognition. The majority of individuals with BBS experience developmental disabilities ranging from mild cognitive impairment or delayed emotional development to severe mental and psychiatric disorders [19]. The frequency of neuropsychiatric disorders and autism in BBS exceeds the incidence rate of these disorders in the general population [20]. However, the role of BBS proteins in cognition remains elusive.

Here, we reveal for the first time, to our knowledge, significant morphological aberrations of dendritic spines in principal neurons of *Bbs* murine models. These changes correlate with impaired performance in contextual and cued fear conditioning tests. While we show that altered synaptic activity or mitochondrial dysfunction are unlikely to cause spine loss, we found that it is correlated with impaired spine membrane receptor signalling, known to be involved in the maintenance of dendritic spines, amongst them IGF-1R and EphB receptor signalling. Moreover, our finding of BBS proteins localisation to the postsynaptic densities (PSDs) of hippocampal neurons prompted us to propose a model for the role of BBS proteins in the structure and function of synapses. While the role of BBS proteins has so far been mainly confined to the functional maintenance of cilia, our data reveal that they play an important role in the development and maintenance of synaptic structures, and suggest that aberrant spine formation and maintenance may contribute to the cognitive impairment phenotype in BBS patients.

## Results

### Loss of Bbs proteins affect principal neuron dendritic morphology

**Fig 1:**
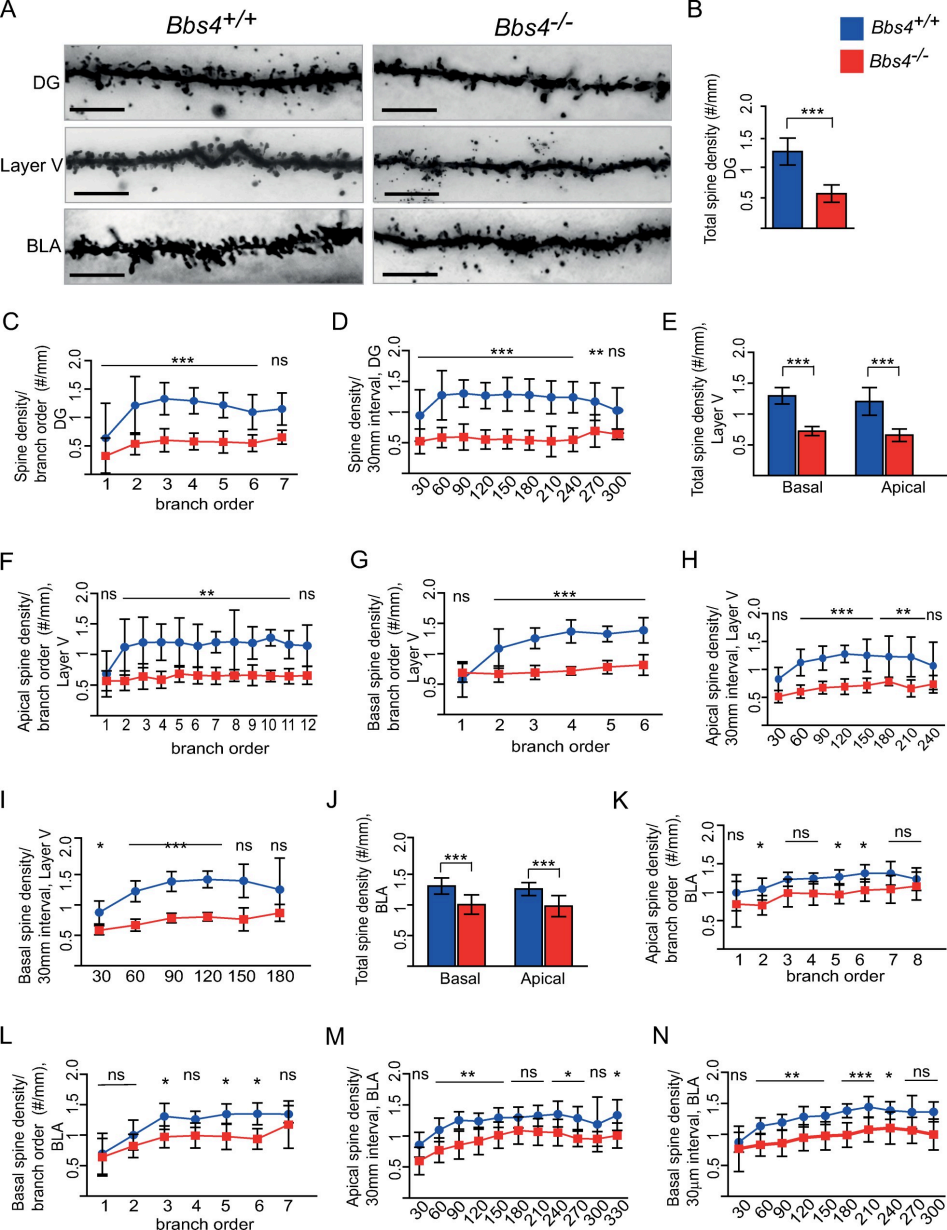
Dendritic spine morphology of DG, BLA, and frontal cortex neurons is affected by the loss of Bbs4 protein. (A) Representative images of Golgi-impregnated DG, BLA, and Layer V pyramidal neurons of P42 *Bbs4*^−/−^ and *Bbs4*^+/+^ mice (100x; scale bar, 5 μm). (B-D) Spine density of DG neurons. (B) Total spine density. (C) Spine density per branch order. (D) Spine density per 30-μm interval. (E-I) Spine density of layer V pyramidal neurons. (E) Total spine density. (F) Spine density in apical dendrites per branch order. (G) Spine density in basal dendrites per branch order. (H) Spine density in apical dendrites per 30-μm interval. (I) Spine density in basal dendrites per 30-μm interval. (J-N) Total spine density of BLA. (J) Total spine density. (K) Spine density in apical dendrites per branch order. (l) Spine density in basal dendrites per branch order. (M) Spine density in apical dendrites per 30-μm interval. (N) Spine density in basal dendrites per 30-μm interval (*N*_mice_/_WT_ = 5; *N*_mice/KO_ = 7, *N*_cells_/_WT_ = 25, *N*_cells_ /_KO_ = 35, mean ± SD, ***P < 0.001; **P < 0.01; *P < 0.05). One-way ANOVA, Tukey post hoc test except for B, E, J, where unpaired *t* test was used. Underlying data are available in S1 Data. *Bbs4*, Bardet-Biedl syndrome 4; BLA, basolateral amygdala; DG, dentate gyrus; KO, knockout; ns, not significant; WT, wild type.

Given that primary cilia are required for the formation of neuronal dendrites [21], we investigated the effect of loss of ciliary Bbs proteins on the dendritic morphology of principal neurons of *Bbs* mouse models. We measured dendritic length, spine count, and spine density of dentate gyrus (DG), basolateral amygdala (BLA), and layer V pyramidal neurons of the frontal cortex using a Golgi-Cox impregnation method. We found that the total spine density was reduced by 55% in DG granule cells of P42 old *Bbs4*^*−/−*^ mice (Fig 1A and 1B and S1 Video). Total spine density on the basal and apical dendrites (further referred to as basal and apical spine density) of layer V neurons was reduced by 55% and 54% (Fig 1A and 1E), respectively, and total basal and apical spine density of BLA neurons was reduced by 23% and 22%, respectively (Fig 1A and 1J). Sholl analysis revealed a significant reduction in spine density of all branches and per 30-μm interval in DG with the exception of the most distal branch and a 300-μm circle in *Bbs4*^*−/−*^ mice (Fig 1C and 1D). Similar Sholl analysis results were found in apical and basal dendrites of Layer V neurons, where dendritic spine density per branch order and per 30-μm interval was affected (Fig 1F–1I). Apical and basal BLA dendrites of *Bbs4*^*−/−*^ mice revealed unequal patterns in spine reduction affecting only a few branches and concentric circles (Fig 1K–1N). A number of dendritic intersections in DG, Layer V, and BLA neurons were not affected when compared with control mice (S1A–S1E Fig). Total dendritic length was reduced by 48% in DG neurons and by 25% in basal dendrites of layer V cortex neurons in *Bbs4*^*−/−*^ mice. Change in the length of apical dendrites of layer V cortex neurons in *Bbs4*^*−/−*^ mice was not statistically significant. BLA apical and basal dendrites showed a statistically significant length reduction of 14% and 19%, respectively (S1F–S1J Fig). Overall, these data show significant aberrations in dendritic morphology in the *Bbs* mouse model.

To determine when the dendritic architecture of *Bbs4*^*−/−*^ DG neurons begins to change, we analysed dendritic length and spine density per branch order and per 30-μm interval at E19.5 and P21. The results of P21 were similar to those obtained at P42: we observed a significant reduction in dendritic length and spine density and no significant changes in a number of dendritic intersections (S2A–S2F Fig). By contrast, at E19.5, the density of dendritic filopodia (dendritic protrusions on developing neurons) in *Bbs4*^*−/−*^ DG neurons were not affected. However, the dendritic length was significantly reduced at E19.5 (S2A and S2G–S2K Fig). Taken together, the *Bbs4*^*−/−*^ murine model shows a progressive decrease in dendritic spine density at P21 (38%) and P42 (55%), but not at late embryonic stages (S2L Fig).

To investigate whether similar dendritic abnormalities can be detected in other *Bbs* models, we analysed the DG dendrite morphology of P21 *Bbs5* and *Bbs1* M390R models. Notably, loss of the Bbs5 protein led to significant reduction in DG dendritic length (34%) and overall spine density (32%) in dentate granule cells of knockout mice (S3A–S3C Fig). Sholl analysis also revealed abnormal spine density in *Bbs5*^−/−^ mice, with a significant spine reduction from the second to fifth branch order and from the 60-μm to 150-μm interval, respectively (S3D–S3F Fig). Interestingly, *Bbs1*^*M390R/M390R*^ was associated with consistent but marginal abnormalities in spinogenesis of DG neurons showing only a 10% reduction in the total spine density. However, dendritic length was not affected (S3A and S3G–S3K Fig). This finding is in agreement with our clinical observations that *BBS1 M390R* patients have the mildest cognitive phenotype.

To determine whether specific subtypes of spines were overrepresented on DG neurons of *Bbs4*^*−/−*^ mice, we analysed spines based on their size and shape (S4A Fig) [22]. We observed that total spine count was reduced in all spine subtypes except ‘branched’ spines. However, when the reduction of dendritic length of *Bbs4*^*−/−*^ neurons was taken into account, we found that only the density of ‘thin’ spines was significantly reduced (22%) (S4B–S4E Fig).

### Reduced contextual and cued fear memory but no impairment in anxiety-like behaviour in *Bbs4*^*−/−*^ mice

**Fig 2:**
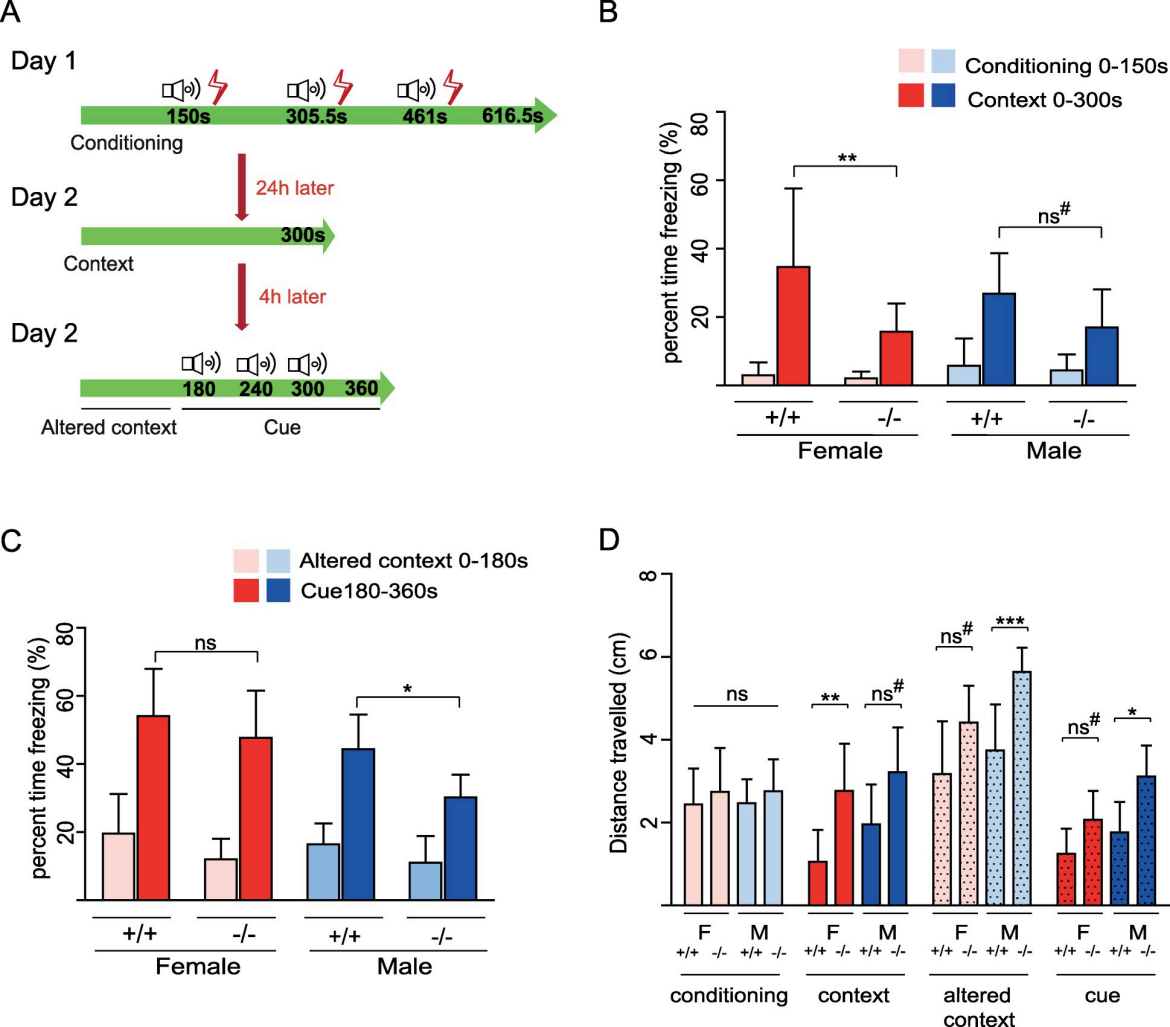
Aberrant fear conditioning behaviour in *Bbs4* knockout mice. (A) Schematic presentation of the contextual and cued fear conditioning test. At Day 1, mice were placed in the fear conditioning chamber for 616.6 seconds. After 150 seconds, a 5-second tone is played, followed by a 0.5-second, 0.5-mA shock. The tone and shock are repeated two more times at 150-second intervals. At Day 2 mice were placed in exactly the same chamber for 300 seconds without tones or shocks. After 4 hours (Day 2), mice are placed in the altered context and left for 180 seconds. At 180 seconds, a 5-second tone is played, which is repeated twice at 60-second intervals. The first 150 seconds of the conditioning trial were used as a baseline for the context data. The first 180 seconds in the altered context were used as the baseline for the cue data. (B) Freezing (%) in the contextual memory test. (C) Freezing (%) in the cue memory test. (D) Distance travelled (cm) in the conditioning test, context test, and cued test (females: *N*_WT_ = 11, *N*_KO_ = 11; males: *N*_WT_ = 13, *N*_KO_ = 12; mean ± SD, ***P < 0.001; **P < 0.01; *P < 0.05). One-way ANOVA, Tukey post hoc test. ^#^It was noted that there was a significant level of reduction of percent time freezing and distance travelled in *Bbs4*^*−/−*^ mice when unpaired, two-tailed *t* test (*P* < 0.05) was used. Underlying data are available in S3 Data. *Bbs4*, Bardet-Biedl syndrome 4; KO, knockout; WT, wild type.

Hippocampus, amygdala, and prefrontal cortex are structures involved in learning, memory, and social interaction. To investigate whether the loss of dendritic spines of DG, BLA, and prefrontal cortex neurons correlates with behavioural changes in *Bbs4*^*−/−*^ mice, we performed a set of behavioural tests. *Bbs* mice are known to develop a number of defects, including visual impairment and obesity [23]. To minimise the effect of these confounding factors, we performed the tests in younger mice (8 weeks). According to the majority of the literature and our own assessments, retinal degenerative changes in this *Bbs4* model begin to develop at 7–8 weeks, making visual impairment unlikely to account for the differences in the test. Similarly, obesity should not confound our results, as at this age there are no weight differences in *Bbs4*^*−/−*^ and *Bbs4*^*+/+*^ mice. To assess fear memory, we performed contextual and cued fear conditioning tests. In the conditioning session at Day 1, freezing behaviour and distance travelled during the first 150 seconds without introducing a conditioned stimulus (tone) and unconditioned stimulus (footshock) were used to evaluate baseline activity in the novel environment of the contextual fear experiment. The loss of Bbs4 did not affect the percentage of freezing or distance travelled in baseline activity of *Bbs4*^*−/−*^ male and female mice. However, after introduction of the paired tone-foot shock stimulus at Day 1, post hoc analysis revealed a significant decrease in the percentage of freezing and an increase in the distance travelled on Day 2 of *Bbs4*^*−/−*^ female mice compared with female control mice. Percentage of freezing and an increase in distance travelled were not statistically significant in male mice (Fig 2A, 2B and 2D).

Altered context at Day 2 before the introduction of the tone was used as a baseline for the cue data. The data revealed that there was no significant change in the percentage of freezing in altered context baseline activity (Fig 2C and 2D). During the cued conditioning session with the tone (Day 2, 180–360 seconds), the percentage of freezing was significantly reduced, and distance travelled increased in *Bbs4*^*−/−*^ male but not in female mice (Fig 2C and 2D). This set of results indicates that loss of Bbs4 protein affects contextual and cued fear memory in a gender-task–dependent manner.

### Miniature excitatory postsynaptic currents amplitude is increased in *Bbs4*^*−/−*^ DG neurons

**Fig 3:**
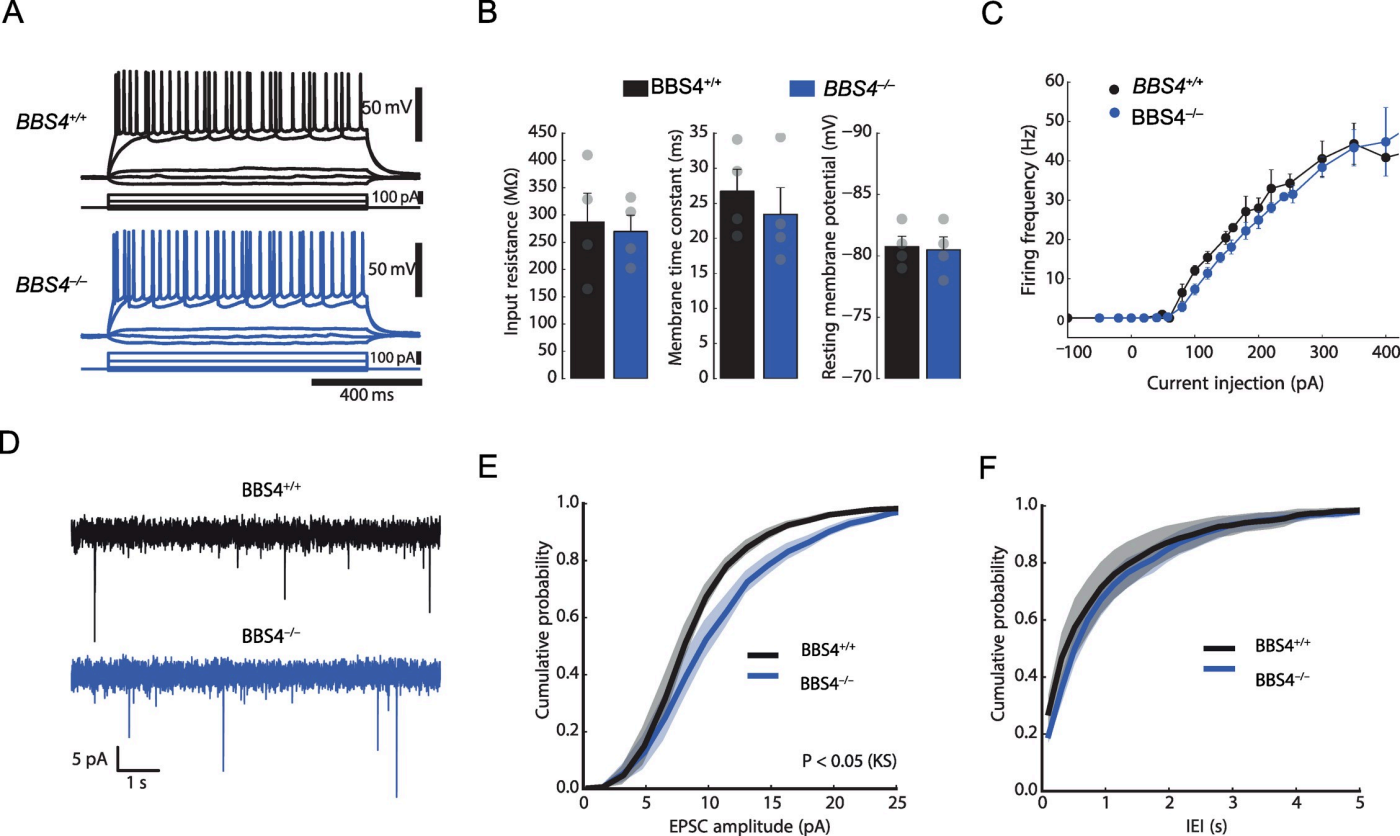
Comparison of electrophysiological properties of hippocampal neurons between *Bbs4*^*−/−*^ *and Bbs4*^*+/+*^ mice in vitro using acute hippocampal slices. (A) Comparison of firing patterns in response to current injections during current clamp recordings from hippocampal granule cells. (B) Bar graphs summarising passive membrane properties. No significant differences were found between *Bbs4*^−/−^ and *Bbs4*^+/+^ mice in input resistance (left), membrane time constant (middle), and resting membrane potential (right). (C) Firing frequency was plotted against current injection amplitudes. No significant differences were found between *Bbs4*^−/−^ and *Bbs4*^+/+^ mice. (D) mEPSCs were recorded from hippocampal granule cells in *Bbs4*^−/−^ and *Bbs4*^+/+^ mice (*N* = 6). (E) Cumulative probability plot comparing mEPSC amplitudes between *Bbs4*^−/−^ and *Bbs4*^+/+^ mice. mEPSC amplitudes in granule cells of *Bbs4*^−/−^ mice are significantly larger (*N* = 6, *P* < 0.05, Kolmogorov-Smirnov test). (F) Cumulative probability plot comparing inter-event intervals (IEIs) of mEPSCs between *Bbs4*^−/−^ and *Bbs4*^+/+^ mice (*N* = 6; *P* = 0.27, Kolmogorov-Smirnov test). Underlying data are available in S3 Data. *Bbs4*, Bardet-Biedl syndrome 4; IEI, inter-event interval; KS, Kolmogorov-Smirnov; mEPSC, miniature excitatory postsynaptic current.

The morphology of dendritic spines is highly dynamic, and their formation and maintenance depend on synaptic function and neuronal activity [24]. To assess synaptic and neuronal function in a Bbs model, we measured intrinsic and synaptic properties of hippocampal granule cells of 3–4-week-old *Bbs4*^*−/−*^ and *Bbs4*^*+/+*^ mice in acute hippocampal slices. We found that intrinsic properties of *Bbs4*^*−/−*^ neurons were unaffected compared with age-matched control littermates (Fig 3A–3C). To evaluate the synaptic properties of granule cells in these two groups, we measured miniature excitatory postsynaptic currents (mEPSCs). Notably, while the frequency of mEPSCs was not different between the two groups, mEPSC amplitudes were significantly larger in *Bbs4*^*−/−*^ neurons (Fig 3D–3F). These data make it unlikely that decreased neuronal activity underlies diminished spine density. On the contrary, the observed increase in mEPSC amplitudes suggests an activation of compensatory mechanisms at the presynaptic and/or postsynaptic sites in response to spine loss.

### IGF-1R downstream signalling is dysregulated in *Bbs4*^*−/−*^ synaptosomes

**Fig 4:**
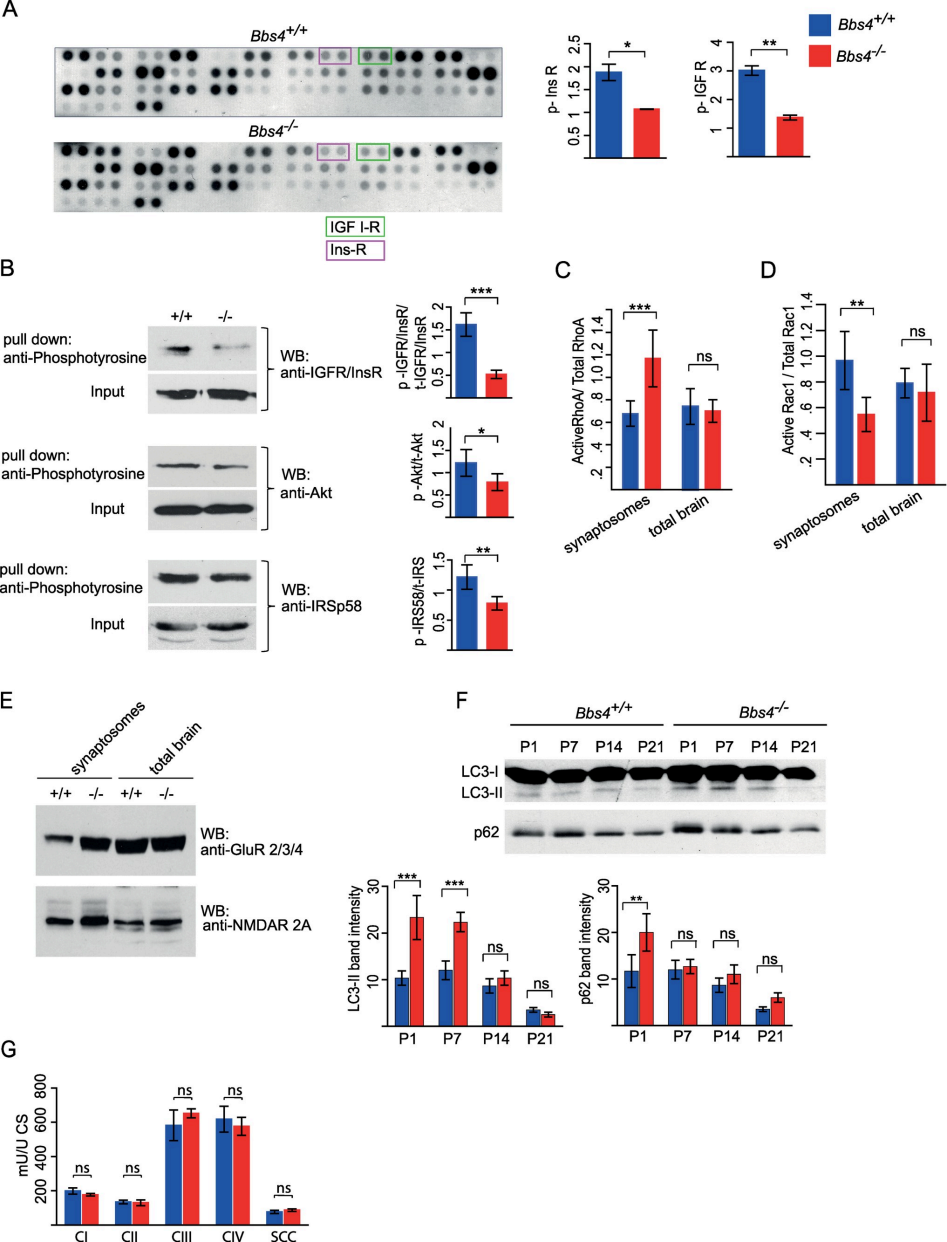
InsulinR/IGF1-R signalling is dysregulated in the synaptosomal fraction of P7 *Bbs4*^*−/−*^ mice. (A) Phospho-RTK array reveals significant decrease in phosphorylation of insulin and IGF1 receptors in *Bbs4*^−/−^ (*N* = 2, mean ± SD). (B) Pull-down analysis shows aberrant IGF-1R downstream signalling. *Bbs4*^−/−^ and *Bbs4*^+/+^ enriched synaptosomal fractions were incubated with mouse anti-phosphotyrosine antibody overnight, followed by incubation with Dynabeads M-280 for 2 hours. Immunoblotting analysis of the proteins eluted from the beads was performed using anti-IGFR/InsR, anti-Akt, and anti-IRS p58 antibodies. Input: the total brain protein fraction before the incubation with anti-phosphotyrosine antibody, which indicates the total level of IGFR/InsR, Akt, and IRS p58 in *Bbs4*^−/−^ and *Bbs4*^+/+^ mice. (C, D) RhoA and Rac1 G-LISA Activation Assays. Levels of activated RhoA (c) and Rac1 (d) were measured in the total brain extracts and enriched synaptosomal fraction of *Bbs4*^−/−^ and *Bbs4*^+/+^ mice (*N* = 3, mean ± SD; unpaired *t* test). (E) Representative image of western blot analysis of NMDA and AMPA receptors levels in the total brain extract and enriched synaptosomal fraction of *Bbs4*^−/−^ and *Bbs4*^+/+^ mice. (F) Representative western blots of autophagy markers LC3-II and p62 in the synaptosomal fractions of *Bbs4*^−/−^ and *Bbs4*^+/+^ mice at P1, P7, P14, and P21 (*N* = 3, mean ± SD). LC3-I is a cytosolic form of LC3. LC3-II is a LC3-phosphatidylethanolamine conjugate (LC3-II), which is recruited to autophagosomal membranes. LC3-II and p62 levels were quantified by measuring western blot band intensities using the Image J programme (*N* = 3, mean ± SD, unpaired *t* test). Housekeeping genes (actin, GAPDH, etc.) could not be used as normalisation controls due to the changes in their gene expression levels in *Bbs4*^−/−^ mice (our unpublished observations). (G) Measurement of oxidative phosphorylation (OXPHOS) complex activities in the whole brain homogenates of *Bbs4*^−/−^ and *Bbs4*^+/+^ mice. Units: mU:U CS; raw data were normalised to citrate synthase; *N* = 4, mean ± SD; ns, not significant; unpaired *t* test. Underlying data are available in S2 Data. AMPAR, alpha-Amino-3-Hydroxy-5-Methyl-4-Isoxazole Propionic Acid receptor; NMDAR, N-methyl-D-aspartate receptor; *Bbs4*, Bardet-Biedl syndrome 4; CI, mitochondria complex I; CII, mitochondria complex II; CIII, mitochondria complex III; CS, citrate synthase, GAPDH, Glyceraldehyde 3-phosphate dehydrogenase; GluR, glutamate receptor; IGF-1R, insulin-like growth factor receptor; InsR, insulin receptor; LC3, microtubule-associated protein 1A/1B-light chain 3; LC3-I, cytosolic form of LC3; LC3-II, LC3-phosphatidylethanolamine conjugate recruited to autophagosomal membranes; OXPHOS, oxidative phosphorylation; RTK, tyrosine kinase receptor; SCC, succinate:cytochrome c oxidoreductase (= complex II + III combined); WB, western blot.

A number of tyrosine kinase receptors (RTKs), including IGF, RET, TrkB, PDGF, and EphB are known to enhance dendritic growth and promote the formation and maintenance of dendritic spines [7,25]. To assess the signalling of RTK in the synaptosomal fractions of *Bbs4*^*−/−*^ and *Bbs4*^*+/+*^ mice, we quantified the phosphorylation level of RTKs using a Phospho-RTK Array. Given that dendritic spine loss occurs between P1 and P21 and to capture the initial signalling changes before potential compensatory mechanisms may start taking place, we used synaptosomal fractions of P7 mice. Synaptosomal fractions from *Bbs4*^*−/−*^ and *Bbs4*^*+/+*^ mice were incubated with the membrane containing immobilised RTK antibodies followed by detection of RTK phosphorylation by a pan anti-phospho-tyrosine antibody. Interestingly, phosphorylation levels of a number of RTKs were altered, including insulin and IGF1 receptors (Fig 4A and S5 Fig). We focused on IGF-1R/insulin receptor (IR) signalling, as it is known to have a profound effect on neuroplasticity in the CNS [7–9]. Pull-down experiments confirmed that phosphorylation of IGF-IR/InsulinR was decreased in the P7 enriched synaptosomal fraction of *Bbs4*^*−/−*^ mice (Fig 4B). Additionally, phosphorylation levels of Akt, a downstream target of canonical IGF signalling, were significantly reduced (Fig 4B). Next, we tested the phosphorylation level of insulin receptor substrate P53 (IRS p58), an adaptor protein that is phosphorylated by IR and IGF-1R [26]. Interestingly, IRS p58 protein has previously been shown to be highly enriched in the PSD of glutamatergic synapses, highlighting the role of this protein in neurons [27]. We found that phosphorylation of IRS p58 was significantly reduced in synaptosomal fractions of P7 *Bbs4*^*−/−*^ mice (Fig 4B). Furthermore, as the activities of IGF-1R and IRS p58 depend on interaction with Rho family GTPases [28, 29], we investigated the activities of Rac1 and RhoA GTPases. We observed that activity of RhoA was increased and, concurrently, Rac1 activity was decreased in the enriched synaptosomal fraction of P7 *Bbs4*^*−/−*^ mice (Fig 4C and 4D). We next assessed whether dysregulation of IGF-1 signalling in Bbs^−/−^ mice affects the levels of N-methyl-D-aspartate (NMDA) and Alpha-Amino-3-Hydroxy-5-Methyl-4-Isoxazole Propionic Acid (AMPA) receptors in the total and synaptosomal fractions of *Bbs4*^*−/−*^ *and Bbs4*^*+/+*^ mice by western blotting. We observed a significant increase in the level of NMDA and AMPA receptors in the synaptosomal fraction of P7 *Bbs4*^*−/−*^ mice, whereas no changes in the receptors’ levels in the total brain fraction were detected (Fig 4E). These data are consistent with our previous findings of increased mEPSC amplitudes in *Bbs4*^*−/−*^ neurons (Fig 3E), suggesting a compensatory mechanism in response to spine loss.

One of the possible mechanisms of dendritic spine pruning is macroautophagy [18], a process that is tightly regulated by IGF-1R signalling and small GTPases. To test whether autophagy is dysregulated in our *Bbs* model, we analysed the level of autophagy markers LC3-II and p62 in the enriched synaptosomal fractions isolated from *Bbs4*^*−/−*^ and *Bbs4*^*+/+*^ mice brains at different postnatal stages. We observed a significant increase in LC3-II level at P1 and P7 of *Bbs4*^*−/−*^ mice (Fig 4F). Given the widely recognised notion that the level of p62 correlates inversely with autophagy, it was unexpected to see an increase in p62 in P1 synaptosomes in our experiment. However, it is in line with reports that p62 levels can be up-regulated during high autophagic flux due to a multifunctional role for p62 [30]. To exclude mitochondrial dysfunction and oxidative stress as triggers of autophagic induction [31], we assessed the activities of respiratory chain complexes I, II, III, IV and succinate:cytochrome c oxidoreductase (SCC; complex II and III combined) in total brain homogenates of P7 *Bbs4*^*−/−*^ and *Bbs4*^*+/+*^ mice. Oxidative phosphorylation (OXPHOS) complex activities were determined, and the results were normalised to the activity of citrate synthase (CS). We found no significant differences in the activities of OXPHOS between *Bbs4*^*−/−*^ and *Bbs4*^*+/+*^ mice (Fig 4G), thus ruling out mitochondrial dysfunction as a cause of autophagy in BBS. Together, our findings suggest that aberrant IGF-1 signalling may lead to dysregulation of various cellular pathways that are known to control dendritic spine morphology and plasticity.

### Synaptic localisation of BBS proteins

The role of Bbs proteins in the regulation of primary cilia has been recently broadened by studies showing that Bbs proteins are involved in microtubular stabilisation, actin remodelling, transcriptional regulation, and endosomal trafficking [16,17,32]. Taking into account this broad spectrum of Bbs functions as well as our current results elaborating the role of Bbs, such as reduction in dendritic spine density along with aberrant synaptic IGF receptor signalling and altered neurotransmitter receptor levels (NMDA and AMPA), we hypothesised that Bbs proteins may play a vital role in neuronal synapses. Re-evaluation of our earlier mass spectrometric analyses of synaptosomal [33,34] and crude synaptosomal fractions [35] of the rat cortex, dorsal striatum, and DG revealed the presence of Bbs1, Bbs2, Bbs4, Bbs5, Bbs7, and Bbs10 proteins (S1 Table).

**Fig 5:**
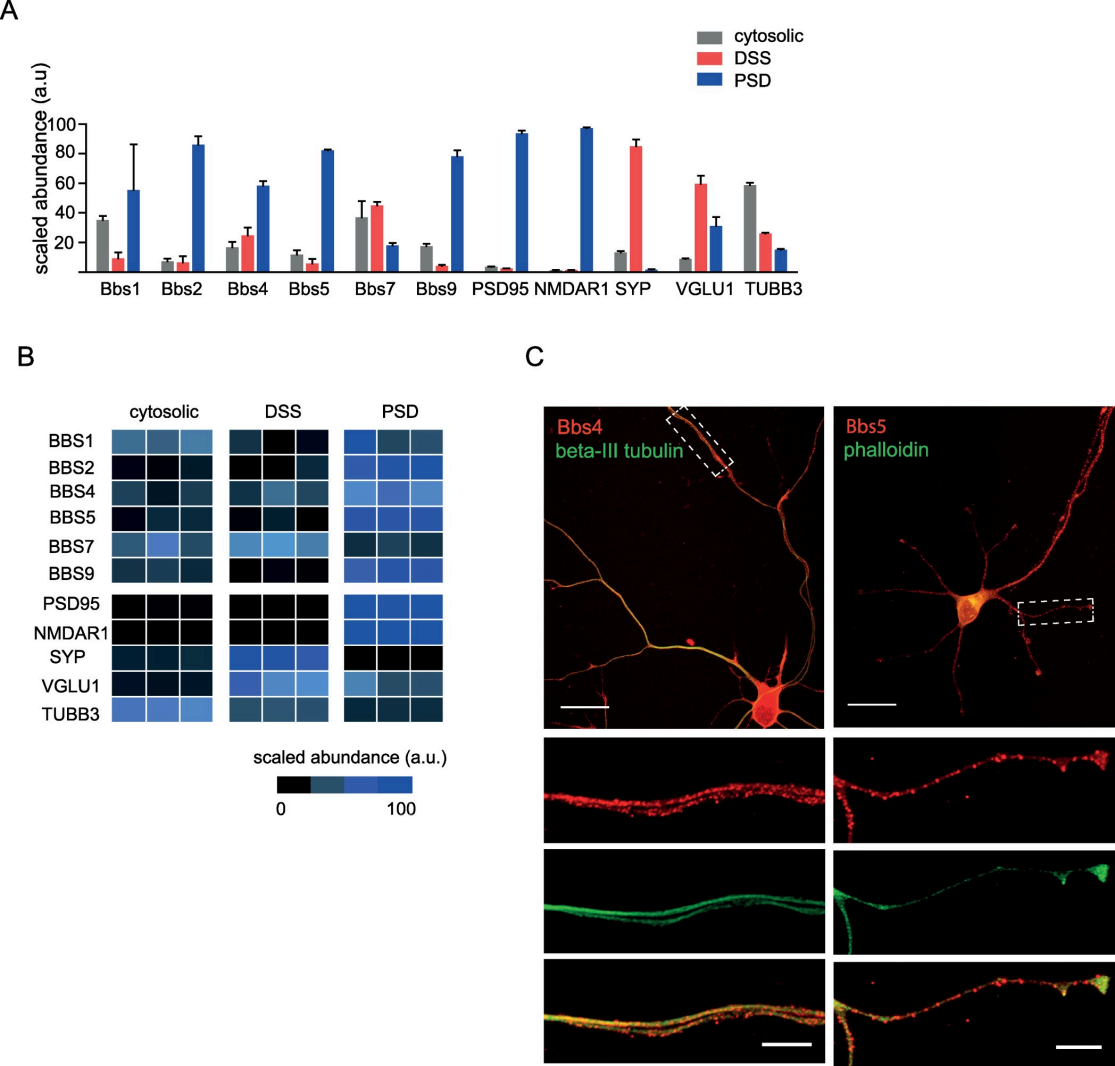
Biochemical and immunocytochemical profiling of BBS proteins. (A) Proteomic profile of the BBS proteins in biochemical fractions using nano-LC-MS/MS analysis. Protein levels of Bbs proteins and synaptic markers were estimated by label-free LC-MS analyses from following biochemical fractions of the rat hippocampi: cytosolic, detergent-soluble synaptosomal preparation (DSS, pre-synapse enriched), and postsynaptic density preparation (PSD). Protein levels of the presynaptic (VGLU1, SYP) and postsynaptic (NMDAR1, PSD95) protein markers are enriched in DSS and PSD preparations, respectively. (B) The protein abundances illustrated in the heat map are obtained from the total MS1 peptide intensities scaled to the mean of all the samples. Protein levels of the presynaptic (VGLU1, SYP) and postsynaptic (NMDAR1, PSD95) protein markers are enriched in DSS and PSD preparations, respectively. (C) Representative image of immunolabelling of Bbs proteins. Cultured mouse hippocampal neurons at low density were immunolabelled with Bbs4 and Bbs5 antibodies (red), phalloidin (green), and beta-III tubulin (green) after 6 days in vitro (DIV6). Scale bar, 20 μm (top panel) and 5 μm (bottom panels). Underlying data are available in S4 Data. BBS, Bardet-Biedl syndrome; DIV6, six days in vitro hippocampal culture; DSS, detergent-soluble synaptosomal; LC-MS/MS, liquid chromatography- tandem mass spectrometry; MS, mass spectrometry; NMDAR1, N-methyl-D-aspartate receptor 1; PSD, postsynaptic density; SYP, synaptophysin; TUBB3, β-tubulin III encoded by TIBB3 gene; VGLU1, vesicular glutamate transporter 1.

To elaborate on synaptic localisation of BBS proteins biochemically, we enriched the cytosolic, detergent-soluble synaptosomal (DSS; pre-synapse enriched), and PSD fractions of synaptosomal preparations from adult rat hippocampi using a previously described method (S6 Fig) [36]. Label-free MS1 intensity-based LC-MS quantitation revealed a high abundance of Bbs1, Bbs2, Bbs5, and Bbs9 proteins in the PSD fraction, whereas Bbs7 was present mostly in the cytosolic fraction (Fig 5A and 5B). A low level of Bbs4 protein was also unambiguously identified in the PSD fractions (Fig 5A and 5B). Immunofluorescence analysis of Bbs4 and Bbs5 localisation confirmed the presence of Bbs punctae throughout the entire dendritic tree of mouse dissociated hippocampal neurons (Fig 5C and S7A–S7C Fig). Collectively, these data clearly indicate the presence of Bbs proteins in neuronal processes and PSDs.

## Discussion

Our study reveals a previously unknown role for BBS proteins in neuronal function. We found significant morphological changes in dendritic spines and dendritic length in various brain regions of *Bbs* mouse models. Several studies have shown an association of neurodevelopmental and neuropsychiatric disorders with morphological and physiological alterations in dendritic spines [2,3], which prompted us to speculate that dysfunction of dendritic spines may contribute to the cognitive deficits in BBS patients. Our data provide several associative links between perturbed spine integrity and cognitive deficits in BBS: first, we show that *Bbs4*^*−/−*^ mice display reduced contextual and cued fear memory. Second, loss of spines occurs in a spine subtype-dependent manner, affecting mostly ‘thin’ spines, which are thought to be specifically involved in learning processes but not in memory maintenance [37]. This finding is in agreement with our clinical observation that learning difficulties in BBS patients are more prevalent than memory deficits (our observational data from the United Kingdom BBS clinic). Finally, we found that the functional loss of different Bbs proteins appears to affect spines to different degrees: for example, the *Bbs1 M390R* missense mutation has only a marginal effect on the spine density, whereas loss of Bbs4 protein causes 55% reduction in spine density. Together, these data support the notion that dendritic spine aberrations may be an essential contributing factor to cognitive deficits in BBS.

Loss of dendritic spines may be attributed to a number of mechanisms, including reduced synaptic activity, mitochondrial dysfunction, and/or aberrant spine membrane receptor signalling. As intrinsic properties and mEPSC frequencies of Bbs4^−/−^ neurons were unaffected, it is unlikely that reduced neuronal activity is the cause of reduced spine density. On the contrary, the increase in mEPSC amplitudes observed in Bbs4^−/−^ neurons suggests that compensatory mechanisms are invoked in response to the spine loss. Similarly, we did not find any mitochondrial dysfunction in *Bbs4*^*−/−*^ brains. Strikingly, we found that signalling of several RTKs was affected in *Bbs4*^*−/−*^ mice, including IGF-1R and ephrin B2 receptor signalling. A number of studies have reported a role for IGF-1R and ephrin B2 receptor signalling in growth, formation, maintenance, and stabilisation of dendrites and dendritic spines [7,38]. Our present work also shows aberrant downstream cascades, including increased autophagy, reduced phosphorylation of IRS p58, and aberrant activity of the small GTPases Rac1 and RhoA in Bbs4^−/−^ synaptosomal fractions. All these events are known to have a diminishing effect on spine density [7,8,39]. Thus, our data provide a potential link between aberrant spine membrane receptor signalling and loss of spines in *Bbs*.

How do ciliary Bbs proteins affect dendritic spine signalling and, eventually, plasticity? It has previously been shown that neuronal cilia are vital for neuronal migration, adult neurogenesis, elongation of dendrites, and memory [21,40–42]. However, the role of ciliary BBS proteins in these processes, as well as in spine homeostasis, has not been studied yet. Thus, our findings of aberrant spine formation in *Bbs* mouse models raise questions about possible mechanisms mediating the effect of cilia and Bbs proteins on spine homeostasis and plasticity. Whether dysfunction of primary cilia signalling resulting from a loss of functional BBS proteins is sufficient to explain aberrant spine formation is unclear, as more complex mechanisms may be involved. The presence of Bbs proteins in the PSD of spines suggests a direct synaptic function of Bbs proteins, in addition to their role in cilia signalling, and therefore favours a more complex mechanism in which the cross talk between ciliary and synaptic BBS proteins is required for normal spine structure and function. The localisation of Bbs proteins to neuronal synapses is in line with previous studies showing a localisation of Bbs proteins outside of primary cilia, such as a nuclear localisation of Bbs7 protein [16] or a re-localisation of Bbs4 protein to immunological synapses in response to contact with antigen-presenting cells (https://is.cuni.cz/webapps/zzp/download/120312678).

**Fig 6:**
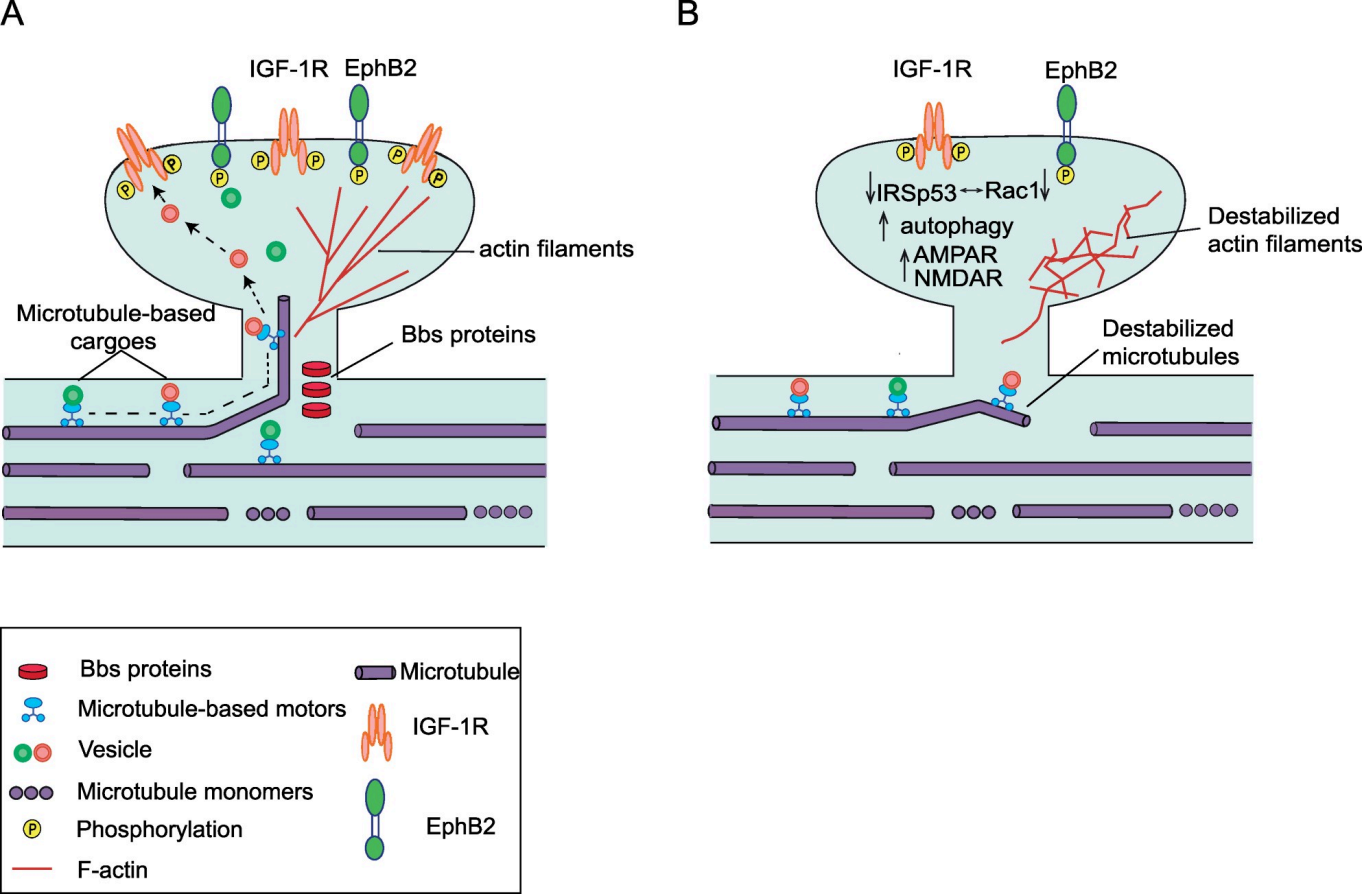
Working model of the role of Bbs proteins in synapses. This simplified schematic is based on (i) the data shown in this paper, (ii) the previously described role of Bbs proteins in primary cilium, and (iii) known dendritic spine homeostasis. It illustrates possible mechanisms by which Bbs proteins may impact synapse plasticity. (A) In the presence of Bbs proteins at the dendritic shafts, microtubules are anchored, facilitating receptor transport to the plasma membrane of the spines. Bbs proteins may also be involved in the transport of the receptors in and out of the spine membrane. (B) In the absence of Bbs proteins the microtubule anchoring and receptor sorting may be destabilised. The receptor transport to the spines becomes dependent on other transport mechanisms, e.g., exocytosis of vesicles in the dendritic shafts or a myosin-based transport [46]. This may result in reduction of the spine membrane receptor abundance (e.g., IGF-1R). This may trigger a number of downstream events, leading to destabilisation of actin filaments and eventually dendritic spines. AMPAR, alpha-Amino-3-Hydroxy-5-Methyl-4-Isoxazole Propionic Acid receptor; Bbs, Bardet-Biedl syndrome; EphB2, ephrin B 2; IGF-1R, insulin-like growth factor; NMDAR, N-methyl-D-aspartate receptor.

Interestingly, comparison between primary cilia and dendritic spines highlights remarkable parallels in their protein composition, membrane domain architecture, and the dynamic nature of their assembly and disassembly [43]. Collectively, it is tempting to speculate that BBS proteins might have similar functions in both structures. Taking also into account that Bbs proteins are known to regulate microtubule assembly and actin reorganisation [18,32], we propose a ‘working hypothesis’ in which BBS proteins are localised to the dendritic spines, where they stabilise microtubule polymerisation (invasions) into dendritic spines, thus facilitating the transport of the signalling receptors to the spine membrane (Fig 6). Further studies are needed to unveil the exact molecular mechanisms of how cilia and ciliary BBS proteins might be involved in the control of receptor trafficking and microtubule organisation in the dendrites and to establish the exact function of synaptic BBS proteins. Potential cilia-dendritic cross talk has recently been discussed [44], suggesting that restoration of 5-hydroxytryptamine, 5HT (5-HT_6_) receptor into the primary cilia of null mutant neurons lengthens both primary cilia and dendrites.

Although it was not investigated in this work, our data may be pivotal in understanding the known interaction between BBS proteins and the disrupted-in-schizophrenia 1 (DISC1) protein, the disruption of which can result in a wide a range of psychiatric conditions, including schizophrenia, bipolar disorder, and major depression [45]. As loss of spines is relevant to many brain disorders including neurodegeneration, probing the synaptic role of BBS proteins will contribute to a deeper understanding of the aetiology of these disorders.

## Methods

### Mice and ethics statement

Animal maintenance, husbandry, and procedures were defined and controlled by the Animal (Scientific Procedures) Act 1986. All animal experiments were carried out under personal and project licences granted by the Home Office (PIL No. 70/7892 and 70/7833) in compliance with Biological Services Management Group and the Biological Services Ethical Committee, UCL, London, UK. Mice were group-housed in IVC cages and were kept on a 12-hour light-dark cycle with ad libitum access to food and water.

The *Bbs1* M390R knock-in model was purchased from Jackson Laboratory, Bar Harbor, ME, *Bbs4* gene trap model was received as a part of previous collaborative work [47], and targeted knockout C57BL/6NTac-Bbs5^tm1b(EUCOMM)Wtsi^/H strain model was received from MRC Harwell as part of the International Mouse Phenotyping Consortium. *Bbs1* and *Bbs4* mice were backcrossed with C57BL/6Ntac strain for five generations to keep the background consistent with *Bbs5* mice obtained from MRC Harwell.

### Dendritic spine analyses

The brains were stained by commercially available Golgi-Cox impregnation kit (NeuroDigiTech). The coronal sections were prepared that covered the anterior-to-posterior axis of the brain, and the regions of interest (ROIs) (DG, BLA, and LV pyramidal neurons) were chosen and analysed using a stereology-based software called NeuroLucida, v10 (Microbrightfield, VT), installed on a Dell PC workstation that controlled Zeiss Axioplan 2 image microscope with Optronics MicroFire CCD camera (1600 × 1200), motorised X-, Y-, and Z-focus for high-resolution image acquisition and digital quantitation. Five or six neurons per ROI were chosen for the analyses. The sampling process was conducted as follows: the investigators (i) previewed the entire rostro-caudal axis of ROI with low-magnification Zeiss objectives (10x and 20x), (ii) compared and located those with the fewest truncations of distal dendrites as possible under high-magnification Zeiss objectives (40x and 63x), and then (iii) used a Zeiss 100x objective with immersion oil to perform 3D dendritic reconstruction, followed by continuous counting of spines throughout the entire dendritic trees. The criteria for selecting candidate neurons for analyses were based on (i) visualisation of a completely filled soma with no overlap of neighbouring soma and completely filled dendrites, (ii) the tapering of most distal dendrites, and (iii) the visualisation of the complete 3D profile of dendritic trees using the 3D display of imaging software. Neurons with incomplete impregnation and/or neurons with truncations due to the plane of sectioning were not collected. Moreover, cells with dendrites labelled retrogradely by impregnation in the surrounding neuropil were excluded. With the systematic registration and digital monitoring, the software was able to accurately record every step of the tracing/contouring and generate a 3D reconstructed dendritic morphology for subsequent spine counting. Automatic navigation of the digital probes with registered x-, y-, and z-coordinates of each 2D image stack enabled creation of a complete 3D neural reconstruction for the dendrograms, spine density, and Sholl analysis. It is noted that only spines orthogonal to the dendritic shaft were readily resolved and included in this analysis, whereas spines protruding above or beneath the dendritic shaft were not sampled (see below). This principle remained consistent throughout the course of analyses. Also, due to inevitable truncations and shrinkage after the impregnation process and optical limitation to resolve most distal dendrites in deep z-axis, underestimates of the actual dendritic lengths and spine numbers would be expected. The above limitations, however, do not affect the comparison of morphological properties between animals. The investigators were blinded to the genotype of the animals.

For spine subtypes analysis, the current study adopted the classification of subtypes of von Bohlen und Halbach [22], including the thin, stubby, mushroom, filopodia, and branched spines. Due to the transient nature of dendritic filopodia (transformation into spines), the sampling of this subtype included all objects that contained small, membranous protrusions detectable on dendritic shafts. The sampling site covered the entire molecular layer of the DG. After completion, the digital profile of spine morphology was transported to PC for a series of breakdown quantitative analysis.

### Mass spectrometric analysis

Hippocampi from fresh Sprague-Dawley rat brains (*n* = 4) were dissected and stored at −80°C. Crude synaptosomes were prepared from tissues according to previously described protocols [48]. Biochemical fractionation of crude synaptosomes into cytosolic, detergent-soluble (pre-synapse-enriched), and post-synapse-enriched synaptosomal fractions were prepared as previously described [36]. Methods for sample preparation for liquid chromatography followed by tandem mass spectrometry (LC-MS/MS) were implemented as previously described [33]. Peptide analyses were performed using a single-shot LC-MS/MS approach with a 4-hour gradient using a Dionex Ultimate 3000 system (Thermo Fisher Scientific) coupled to a Q-Exactive Plus mass spectrometer (Thermo Fisher Scientific, Germany) with LCMS parameters, as described previously [49]. All MS-MS2 spectra were searched against UniProtKB/Swiss-Prot rat protein database version v 2016.04.14. Parameters for protein identification and label-free quantification workflow were based on the Minora algorithm through Proteome Discoverer 2.2 platform, as previously described [49]. Abundances are scaled according to the mean abundance of the samples.

### Electrophysiology

Transverse 300-μm-thick slices were cut from the hippocampi of 3–4-week-old mice with a VT1200 vibratome (Leica Microsystems, Wetzlar, Germany). The animals were anaesthetised with isoflurane added to the inspiration airflow (4%–5%; Abbott, Ludwigshafen, Germany) and killed by decapitation, in accordance with national and institutional guidelines. The slicing solution was sucrose based, containing (in mM) 87 NaCl, 25 NaHCO_3_, 2.5 KCl, 1.25 NaH_2_PO_4_, 75 sucrose, 0.5 CaCl_2_, 7 MgCl_2_, and 10 glucose (equilibrated with 95% O_2_−5% CO_2_). After preparation, slices were kept at 35°C for 30 minutes and then stored at room temperature in artificial cerebrospinal fluid (ACSF) containing (in mM) 125 NaCl, 25 NaHCO_3_, 2.5 KCl, 1.25 NaH_2_PO_4_, 2 CaCl_2_, 1 MgCl_2_, and 25 glucose (equilibrated with 95% O_2_−5% CO_2_).

For electrophysiological experiments, slices were continuously superfused with ACSF. Patch pipettes (4–8 MΩ) were pulled from borosilicate glass tubing with 1.5-mm outer diameter and 0.86-mm inner diameter (Warner, Hamden, USA). The pipettes were filled with a solution containing 130 mM potassium gluconate, 20 mM KCl, 2 mM MgCl_2_, 4 mM K_2_ATP, 0.3 mM NaGTP, 10 mM sodium phosphocreatine, and 10 mM HEPES. The pH was adjusted to 7.3 by adding KOH. Voltage signals and currents were measured with a Multiclamp 700B amplifier (Molecular Devices, Palo Alto, CA), filtered at 5 kHz and digitised at 20 kHz using a Digidata 1550 interface (Molecular Devices, Palo Alto, CA). Data analysis was performed with Stimfit [50]. Membrane potentials were not corrected for liquid junction potentials. All recordings were made at near-physiological temperatures (32–34°C). Spontaneous mEPSCs were recorded at a membrane potential of −80 mV in the presence of 0.5 μM TTX, 1 μM gabazine, and 25 μM D-AP5. For detection and analysis of miniature synaptic events, a template-matching algorithm was used, implemented in Stimfit [50–52].

### Contextual fear conditioning test and cue fear conditioning

Context and cue-dependent fear conditioning experiments were performed using a fear conditioning chamber (bought from Ugo Basile). Mice were trained and tested in a chamber with clear plastic walls and ceiling and a standard grid floor. On Day 1, mice were placed into the fear conditioning chamber for 616.5 seconds. After 150 seconds, a 5-second tone was played, followed immediately by a 0.5-second, 0.5 mA shock. The tone-shock pairing was repeated another two times at 150-second intervals. For the contextual conditioning test, on Day 2, mice were placed in exactly the same arenas as Day 1 for 300 seconds. No tones or shocks were presented. Video tracking was recorded. For the cued conditioning test, on Day 2, 4 hours after the contextual test, mice were placed in an altered context (round arenas, flat bottomed instead of a grid, stripy walls, added vanillin scent around the top, and reduced light for 180 seconds with no tones or shocks. At 180 seconds, a 5-second tone was added, for a total of three tones played at 60-second intervals. Video tracking was recorded, and scoring of freezing behaviour was automatically performed and analysed by Any-Maze software.

### Phospho-RTK array, pull-down experiments, and western blotting

Total brain extracts and enriched synaptosomal fractions from the whole brain were isolated from E19.5 embryos and P1, P7, P14, and P21 postnatal mice using RIPA buffer and Syn-PER Synaptic Protein Extraction Reagent (ThermoFisher Scientific) with protease inhibitors cocktail (ThermoFisher Scientific) and in accordance with standard procedure and the manufacture’s protocol. Protein concentration was determined using Pierce BCA Protein Assay Reagent (ThermoFisher Scientific). Phospho-RTK array (R&D systems) was performed using total brain protein extracts of P7 *Bbs4*^*−/−*^ and *Bbs4*^*+/+*^ mice and in accordance with manufacture protocol. For pull-down experiments, enriched synaptosomal fractions of *Bbs4*^*−/−*^ and *Bbs4*^*+/+*^ were incubated with mouse anti-phosphotyrosine (BD Transduction) antibodies overnight at 4°C, incubated with sheep anti-mouse IgG Dynabeads M-280 (ThermoFisher Scientific) for 2 hours at 4°C, and analysed by Western blotting using anti-IGFR/InsR (Cell Signaling), anti-Akt (Cell Signaling), anti-IRS58 (Abcam) antibodies. The levels of AMPARs and NMDARs in total brain and enriched synaptosomal fractions were analysed using anti-GluR 2/3/4 (Cell Signaling), anti-NMDA subunit 2A (Life Technologies) antibodies, and standard western blotting procedure. The intensities of the bands were analysed by ImageJ software.

The activities of Rac1 and RhoA small GTPases in the total brain and enriched synaptosomal fractions were measures using G-LISA RhoA activation assay (Cytoskeleton, BK124) and G-LISA Rac1 activation assay (Cytoskeleton, BK128). The activity of RhoA was normalised to the total level of RhoA (Cytoskeleton, BK150).

### Immunofluorescence and image acquisition

Primary hippocampal cultures were prepared from E16.5 C57BL/6Ntac mouse embryos and cultured at medium density (100 cells per mm^2^) in N2/B27 medium for 6 days in vitro (DIV6) [53]. Dissociated neurons were fixed with 4% formaldehyde, permeabilised with cold 100% methanol, blocked with 1% horse serum, and incubated with primary antibodies at RT for 1 hour. Primary antibodies against tubulin (Tuj-1) (Chemicon), Bbs5 and Bbs4 (ProteinTech), and Alexa Fluor phalloidin 488 (Invitrogen) were used. Secondary antibodies Alexa 488 and Alexa 568 were from Invitrogen. The images were taken using Zeiss LSM 880 upright confocal microscope with Airyscan, 63x/NA1.4 Plan Apo Oil lens.

### Mitochondria function

Mitochondrial respiratory chain enzyme and CS activities in 600-g supernatants of whole brain homogenates (10% w/V in 0.25 M sucrose, 2 mM EDTA, 10 mM K_2_HPO_4_/KH_2_PO_4_, pH 7.4) were measured following previously described procedures [54] and references therein on a Konelab 20XT spectrophotometric analyzer (ThermoFisher Scientific).

### Statistical analyses

The investigators were blinded to the identities of the samples. Analysis with two groups were performed using an unpaired, two-tailed Student *t* test. Analysis with more than two groups and with one variable were performed using one-way ANOVA and Tukey post hoc tests. Kolmogorov-Smirnov test was used to determine the cumulative distribution function of continuous random variables such as frequency and amplitudes of mEPSCs.

## Supporting information

S1 Table Published and current proteomics data in which BBS proteins were identified. **LC-MS-based proteomic analyses of rat synaptosomal and membrane preparations.** BBS, Bardet-Biedl syndrome; LC-MS, liquid chromatography-mass spectrometry.
(TIF)

S1 Video Reconstructed hippocampal *Bbs4^−/−^* neurons show a significant reduction in total dendritic spines when compared with *Bbs4^+/+^* neurons. For 3D neuron reconstruction (Neurolucida, MBF), the image stacks of representative neurons per group were selected in order to align serial contoured objects, including the soma, apical and basal dendrites, and associated spines. The module ‘3D visualization’ of Neuroludica software was used in order to automatically generate 3D visualisation of representative neurons. *Bbs4*, Bardet-Biedl syndrome.
(MP4)

S1 Fig **(A-E) Sholl analysis of DG, BLA, and Layer V frontal cortex neurons of P42 *Bbs4* mice.** Frequency of intersections per 30-μm interval in DG (A), apical dendrites of layer V pyramidal neurons (B) and basal dendrites of layer V pyramidal neurons (C), apical dendrites of BLA (D), and basal dendrites of BLA (E) (*N*_WT_ = 5; *N*_KO_ = 7, mean ± SD, *P < 0.05); one-way ANOVA, Tukey post hoc test. (F-J) Dendritic length of DG, BLA, and layer V neurons (biological samples: *N*_WT_ = 5; *N*_KO_ = 7; total number of analysed cells: *N*_WT_ = 25; *N*_KO_ = 35, for DG; biological samples: *N*_WT_ = 3; *N*_KO_ = 3; total number of analysed cells: *N*_WT_ = 15; N_KO_ = 15 for BLA and LV) mean ± SD, *P < 0.05; unpaired *t* test. Underlying data are available in S1 Data. *Bbs4*, Bardet-Biedl syndrome; BLA, basolateral amygdala; DG, dentate gyrus; KO, knockout; WT, wild type.
(TIF)

S2 Fig Dendritic spine density is reduced in DG neurons of *Bbs4^−/−^* mice at P21 but not at E19.5. (A) Representative images of Golgi-Cox impregnated dentate granule (DG) of *Bbs4*^*−/−*^ and *Bbs4*^*+/+*^ mice at E19.5 and P21 (100x; scale bar, 5 μm). (B-F) Analysis of DG neuron morphology at E19.5. (B) Total spine density. (C) Dendritic length. (D) Spine density per branch order. (E) Spine density per 30-μm interval. (F) Frequency of intersections per 30-μm interval. (G-K) Analysis of DG neuron morphology at P21. (G) Total spine density. (H) Dendritic length. (I) Spine density per branch order. (J) Spine density per 30-μm interval. (K) Frequency of intersections per 30-μm interval. (L) Schematic representation of spine loss in hippocampal neurons of *Bbs4*^*−/−*^ at different time points (*N*_mice/WT_ = 3; *N*_mice/KO_ = 3, mean ± SD, ***P < 0.001; **P < 0.01; *P < 0.05). One-way ANOVA, Tukey post hoc test except for B, C, H, and I, for which unpaired *t* test was used. Underlying data are available in S2 Data. *Bbs4*, Bardet Biedl syndrome 4; DG, dentate gyrus; KO, knockout; WT, wild type.
(TIF)

S3 Fig Defects in dendritic morphology of DG granule cells of P21 *Bbs5* knockout and *Bbs1* M390R knock-in models. (A) Representative images of Golgi-impregnated DG granule cells of *Bbs5* and *Bbs1* M390R models. (B-F) Analysis of DG granule cells of *Bbs5*^*−/−*^ *and Bbs5*^*+/+*^ mice. (B) Total spine density. (C) Dendritic length. (D) Spine density per branch order. (E) Spine density per 30-μm interval. (f) Frequency of intersections per 30-μm interval. (G-K) Analysis of DG granule cells of *Bbs1*^M390R/M390R^ and *Bbs1*^+/+^ mice. (G) Total spine density. (H) Dendritic length. (I) Spine density per branch order. (J) Spine density per 30-μm interval. (K) Frequency of intersections per 30-μm interval. Biological samples: *N*_WT/*Bbs5*_ = 5; *N*_KO/*Bbs5*_ = 5; total number of analysed cells: *N*_WT/*Bbs5*_ = 22; *N*_KO/*Bbs5*_ = 25; *N*_WT/*Bbs1*_ = 3; *N*_KO/*Bbs1*_ = 3; total number of analysed cells: *N*_WT/*Bbs5*_ = 15; *N*_KO/*Bbs5*_ = 15; mean ± SD, *P < 0.05, ***P < 0.01; one-way ANOVA, Tukey post hoc test. Scale bar, 5 μm. Underlying data are available in S2 Data. *Bbs*, Bardet-Biedl syndrome; DG, dentate gyrus; KO, knockout; WT, wild type.
(TIF)

S4 Fig ‘Thin’ spines are abundant on DG neurons of P42 *Bbs4^−/−^* mice. (A) Illustration of dendritic spine subclasses. Adopted from [22]. (B) Total count of ‘thin’, ‘stubby’, ‘mushroom’, ‘filopodia’, and ‘branched’ spines. (C) Total spine density of thin, stubby, mushroom, filopodia, and branched spines. Density is calculated as the number of spines per micrometre of dendrite. Boxed panel shows the reduction in dendritic length. (D) Spine density of ‘thin’ and ‘mushroom’ spines (*N*_mice/WT_ = 3; *N*_mice/KO_ = 3, *N*_cells/WT_ = 15, *N*_cells/KO_ = 15, mean ± SD, ***P < 0.001; **P < 0.01; *P < 0.05); one-way ANOVA, Tukey post hoc test. Underlying data are available in S2 Data. *Bbs4*, Bardet Biedl syndrome 4; DG, dentate gyrus; KO, knockout; WT, wild type.
(TIF)

S5 Fig RTK phosphorylation assay of enriched synaptosomal fractions of P7 *Bbs4^−/−^* and *Bbs4^+/+^* mice. **(a)** An image of **the** Phospho-RTK array with annotation box. **(b)** Quantitative dot-blot analysis reveals significant decrease in phosphorylation of a number of RTKs in P7 of *Bbs4*^−/−^ synaptosomal fraction (*N* = 3, mean ± SD); unpaired *t* test; ImageJ software. Underlying data are available in S2 Data. *Bbs4*, Bardet Biedl syndrome 4; RTK, tyrosine kinase receptor.
(TIF)

S6 Fig Illustration of an experimental workflow. Cytoplasmic and synaptosomal fractions were isolated from the cortices of three Wistar rats. Synaptosomes were further separated into DSS and PSD fractions. A FASP protocol adapted for synaptic membrane proteins is coupled to a gel-free LC-MS to allow analyses of synaptosomal fractions. Database search was performed with search engines against the rat SwissProt protein database. Quantitative information was determined using the software tools, Proteome Discoverer and Isobar. DSS, detergent-soluble synaptosomal; FASP, filter-aided sample preparation; LC-MS, liquid chromatography tandem mass spectrometry; PSD, postsynaptic density.
(TIF)

S7 Fig Validation of the specificity of *Bbs4* and *Bbs5* antibodies. (A) List of published validations of Bbs4 (12766-1-AP) and Bbs5 (14569-1-AP) ProteinTech antibodies used in this study. (B) Total protein extracts of the *Bbs4*^*−/−*^, *Bbs4*^*+/+*^, *Bbs5*^*−/−*^, and *Bbs5*^*+/+*^ mice were immunoblotted with Bbs4 and Bbs5 antibodies as indicated. Approximate molecular weights are listed on the left side. Gapdh was used as the loading control. Wild-type *Bbs4* and *Bbs5* mice showed a specific single band. Western blot with Bbs4 and Bbs5 antibodies did not detect any specific band in *Bbs4* and *Bbs5* knockout mice. (C) Images of *Bbs4*^*−/−*^ and *Bbs5*^*−/−*^ dissociated neurons immunolabelled with anti-Bbs4 and anti-Bbs5 antibodies. *Bbs*, Bardet-Biedl syndrome; Gapdh, Glyceraldehyde 3-phosphate dehydrogenase.
(TIF)

S1 Data Numerical data used in Fig 1B–1N. (XLSX)

S2 Data Numerical data used in Fig 2B–2D. (XLSX)

S3 Data Numerical data used in Fig 3B–3F. (XLSX)

S4 Data Numerical data used in Fig 4A–4D, 4F and 4G. (XLSX)

## Abbreviations

ACSF: artificial cerebrospinal fluid
Akt: protein kinase B (PKB)
AMPA: Alpha-Amino-3-Hydroxy-5-Methyl-4-Isoxazole Propionic Acid
BBS: Bardet-Biedl syndrome
Bbs4: Bardet-Biedl syndrome 4
BDNF: brain-derived neurotrophic factor
BLA: basolateral amygdala
CNS: central nervous system
CS: citrate synthase
DG: dentate gyrus
DISC1: disrupted-in-schizophrenia 1
DSS: detergent-soluble synaptosomal
D1: dopamine receptor
E: embryonic day
EphB: ephrin B
GAPDH: Glyceraldehyde 3-phosphate dehydrogenase
GluR: glutamate receptor
GPR: orphan G protein-coupled receptor
Gpr161: G-protein coupled receptor 161
IEI: inter-event interval
IGF-1R: insulin-like growth factor receptor
IR: insulin receptor
IRS p58: insulin receptor substrate P58
KS: Kolmogorov-Smirnov
LC-MS/MS: liquid chromatography -tandem mass spectrometry
Mchr1: melanin-concentrating hormone receptor 1
mEPSC: miniature excitatory postsynaptic current
mTOR: mammalian target of rapamycin pathway
NMDA: N-methyl-D-aspartate
OXPHOS: oxidative phosphorylation
P: postnatal day
PSD: postsynaptic density
ROI: region of interest
RTK: tyrosine kinase receptor
SCC: succinate:cytochrome c oxidoreductase (= complex II + III combined)
Sstr3: somatostatin receptor type 3
SYP: synaptophysin
TrkB: tyrosine receptor kinase B
TUBB3: β-tubulin III encoded by TIBB3 gene
VGLU1: vesicular glutamate transporter 1
WT: wild type
5-HT6: subtype of 5HT receptor
