# Correction: Loss of Bardet-Biedl syndrome proteins causes synaptic aberrations in principal neurons

**DOI:** 10.1371/journal.pbio.3000520

**Published:** 2019-10-08

**Authors:** Naila Haq, Christoph Schmidt-Hieber, Fernando J. Sialana, Lorenza Ciani, Janosch P. Heller, Michelle Stewart, Liz Bentley, Sara Wells, Richard J. Rodenburg, Patrick M. Nolan, Elizabeth Forsythe, Michael C. Wu, Gert Lubec, Patricia C. Salinas, Michael Häusser, Philip L. Beales, Sofia Christou-Savina

The fourteenth author's name is spelled incorrectly. The correct name is: Patricia C. Salinas.

The following information is also missing from the Funding statement: This study was supported by the MRC (MR/M024083/1) to PCS. The funding statement in full should read:

This study was supported by grants from the Biotechnology and Biological Sciences Research Council (BBSRC) (BB/M020991/1) and New Life Foundation (14-15/20) to SC-S and PLB; ERC (AdG 695709 DENDRITECIRCUITS to MH and StG 678790 NEWRON to CS-H) and the Wellcome Trust (PRF 536070) to MH; MRC (MR/M024083/1) to PCS. Additional support was funded by National Institute for Health Research (GOS ICH). The funders had no role in study design, data collection and analysis, decision to publish, or preparation of the manuscript.

## References

[1] Haq N, Schmidt-Hieber C, Sialana FJ, Ciani L, Heller JP, Stewart M, et al (2019) Loss of Bardet-Biedl syndrome proteins causes synaptic aberrations in principal neurons. PLoS Biol 17(9): e3000414 10.1371/journal.pbio.3000414 31479441 PMC6743795

