# Supplementary figures and images for: Loss of Bardet-Biedl syndrome proteins causes synaptic aberrations in principal neurons
Source: PLoS Biol. 2019 Sep 3;17(9):e3000414. doi: 10.1371/journal.pbio.3000414 (PMC6743795; doi:10.1371/journal.pbio.3000414)

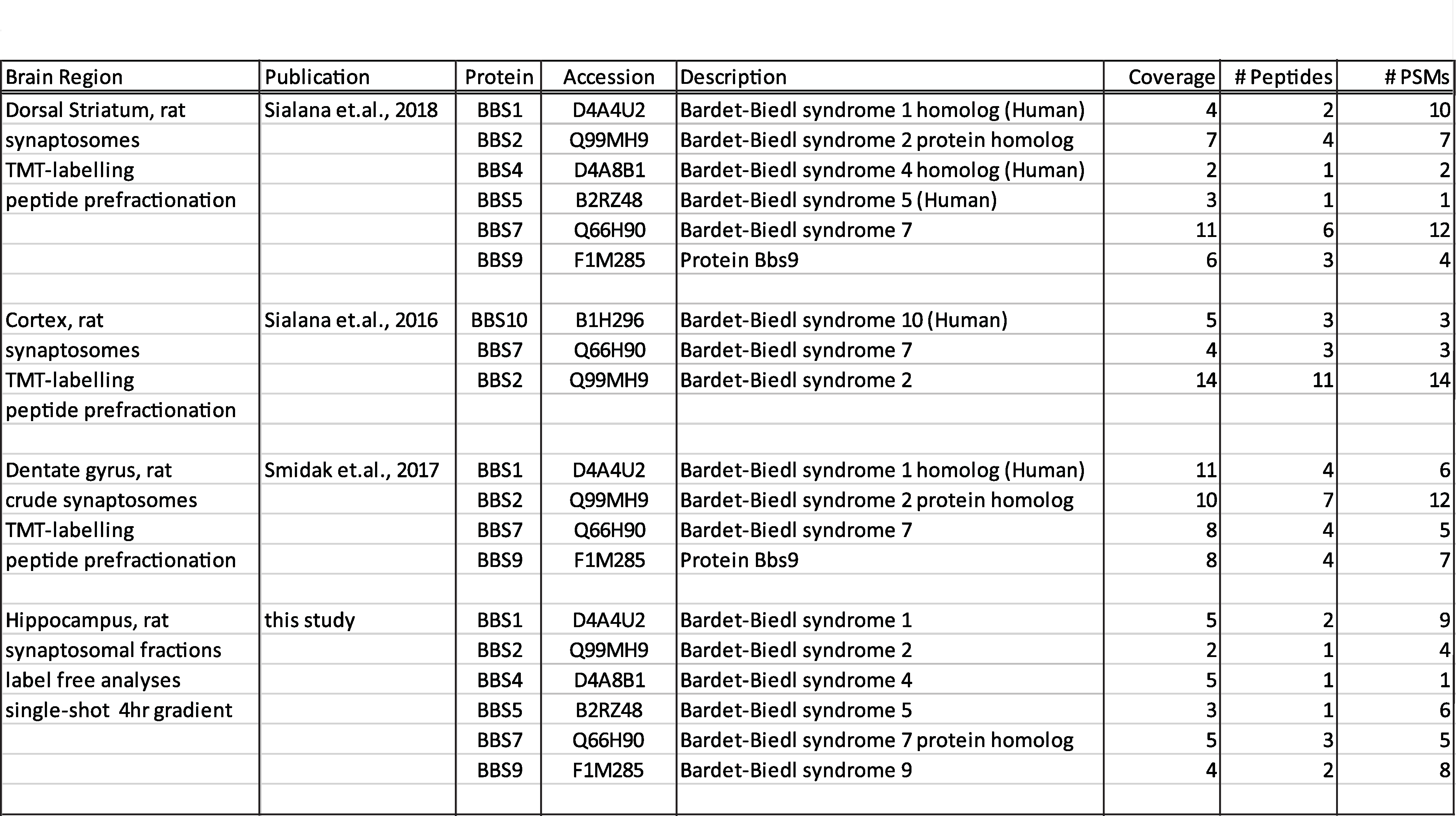

Supplement: S1 Table — LC-MS-based proteomic analyses of rat synaptosomal and membrane preparations. BBS, Bardet-Biedl syndrome; LC-MS, liquid chromatography-mass spectrometry. (TIF) [file pbio.3000414.s001.tif]

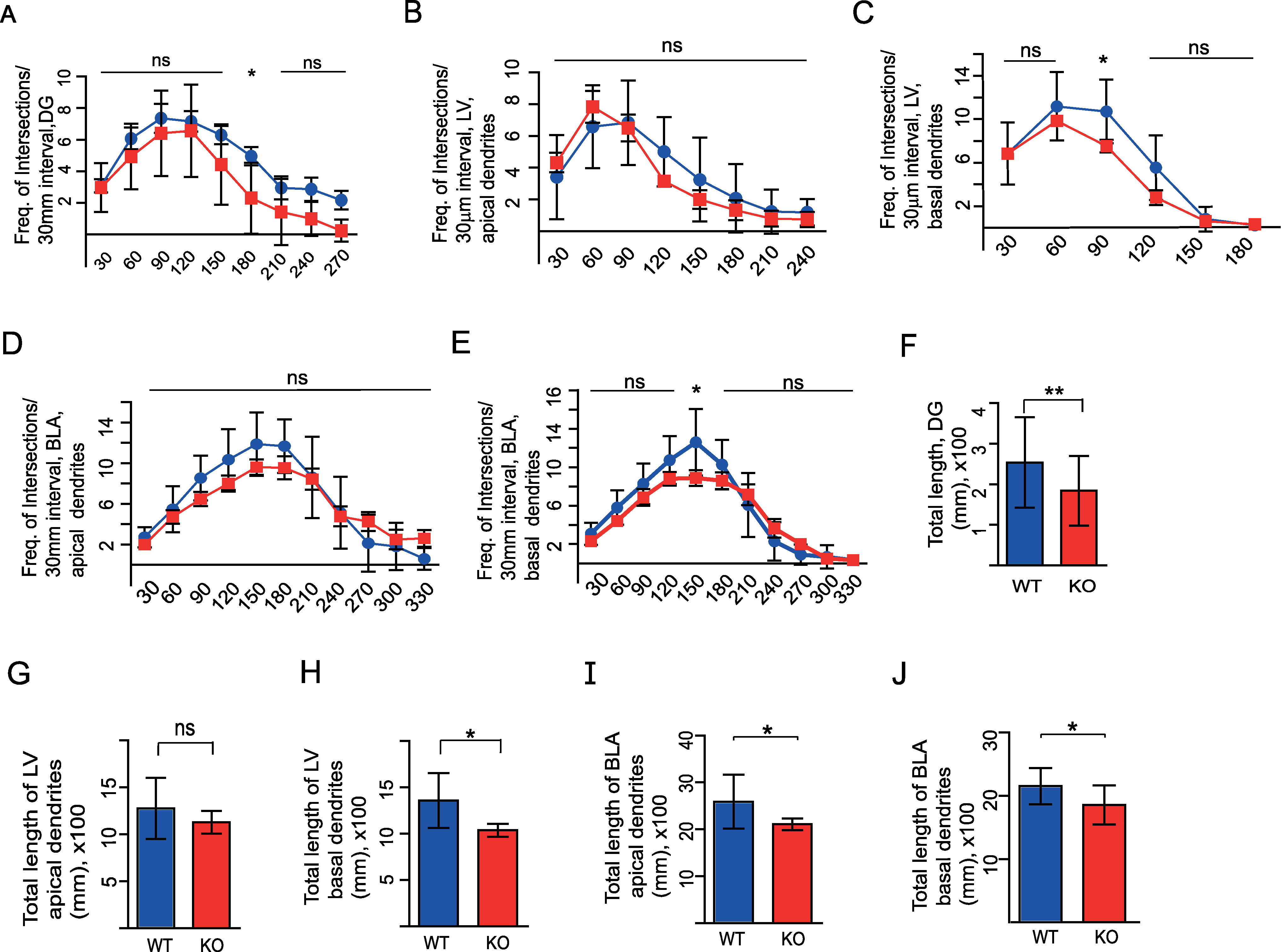

Supplement: S1 Fig — (A-E) Sholl analysis of DG, BLA, and Layer V frontal cortex neurons of P42 Bbs4 mice. Frequency of intersections per 30-μm interval in DG (A), apical dendrites of layer V pyramidal neurons (B) and basal dendrites of layer V pyramidal neurons (C), apical dendrites of BLA (D), and basal dendrites of BLA (E) (NWT = 5; NKO = 7, mean ± SD, *P < 0.05); one-way ANOVA, Tukey post hoc test. (F-J) Dendritic length of DG, BLA, and layer V neurons (biological samples: NWT = 5; NKO = 7; total number of analysed cells: NWT = 25; NKO = 35, for DG; biological samples: NWT = 3; NKO = 3; total number of analysed cells: NWT = 15; NKO = 15 for BLA and LV) mean ± SD, *P < 0.05; unpaired t test. Underlying data are available in S1 Data. Bbs4, Bardet-Biedl syndrome; BLA, basolateral amygdala; DG, dentate gyrus; KO, knockout; WT, wild type. (TIF) [file pbio.3000414.s003.tif]

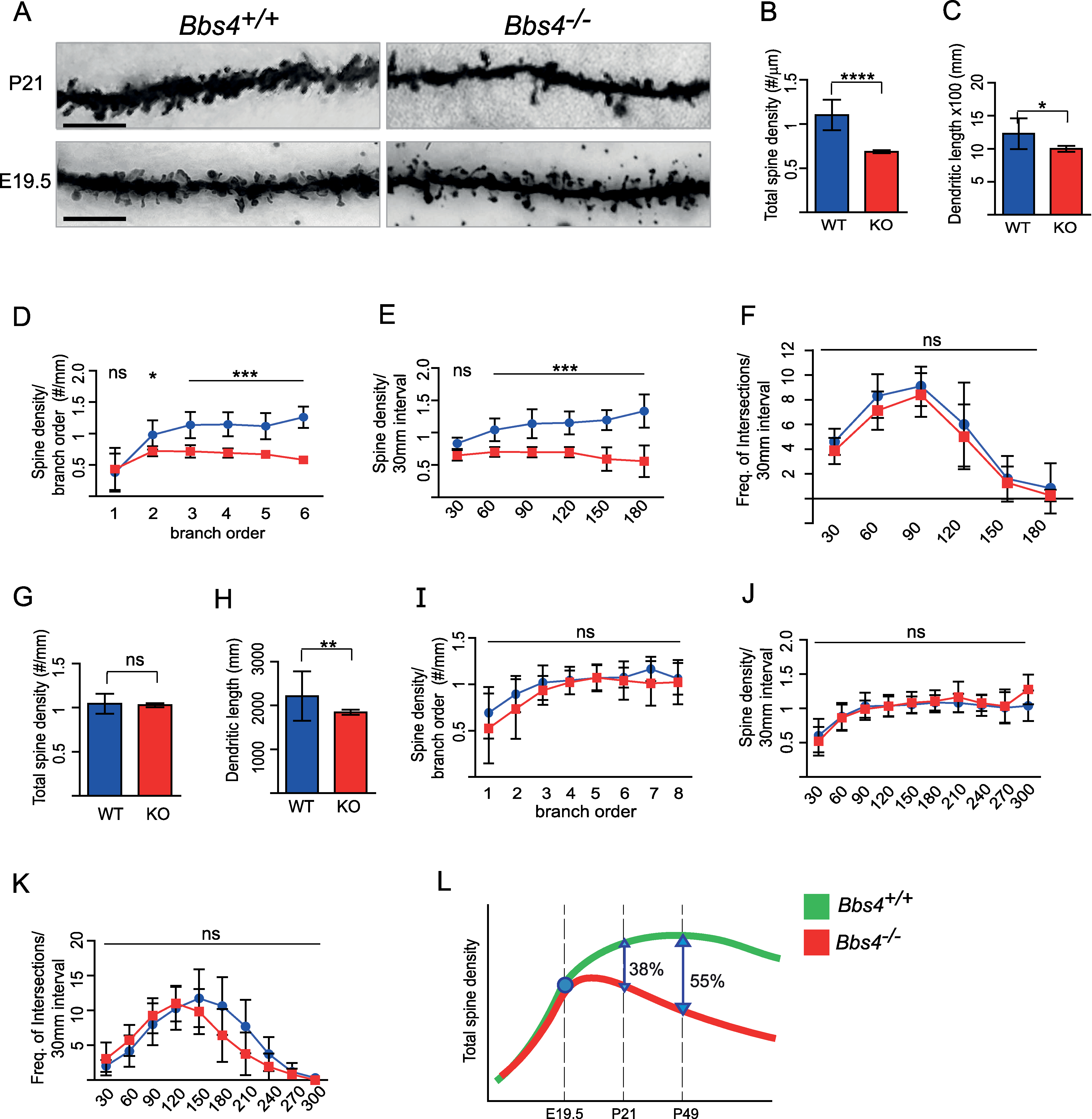

Supplement: S2 Fig — (A) Representative images of Golgi-Cox impregnated dentate granule (DG) of Bbs4−/− and Bbs4+/+ mice at E19.5 and P21 (100x; scale bar, 5 μm). (B-F) Analysis of DG neuron morphology at E19.5. (B) Total spine density. (C) Dendritic length. (D) Spine density per branch order. (E) Spine density per 30-μm interval. (F) Frequency of intersections per 30-μm interval. (G-K) Analysis of DG neuron morphology at P21. (G) Total spine density. (H) Dendritic length. (I) Spine density per branch order. (J) Spine density per 30-μm interval. (K) Frequency of intersections per 30-μm interval. (L) Schematic representation of spine loss in hippocampal neurons of Bbs4−/− at different time points (Nmice/WT = 3; Nmice/KO = 3, mean ± SD, ***P < 0.001; **P < 0.01; *P < 0.05). One-way ANOVA, Tukey post hoc test except for B, C, H, and I, for which unpaired t test was used. Underlying data are available in S2 Data. Bbs4, Bardet Biedl syndrome 4; DG, dentate gyrus; KO, knockout; WT, wild type. (TIF) [file pbio.3000414.s004.tif]

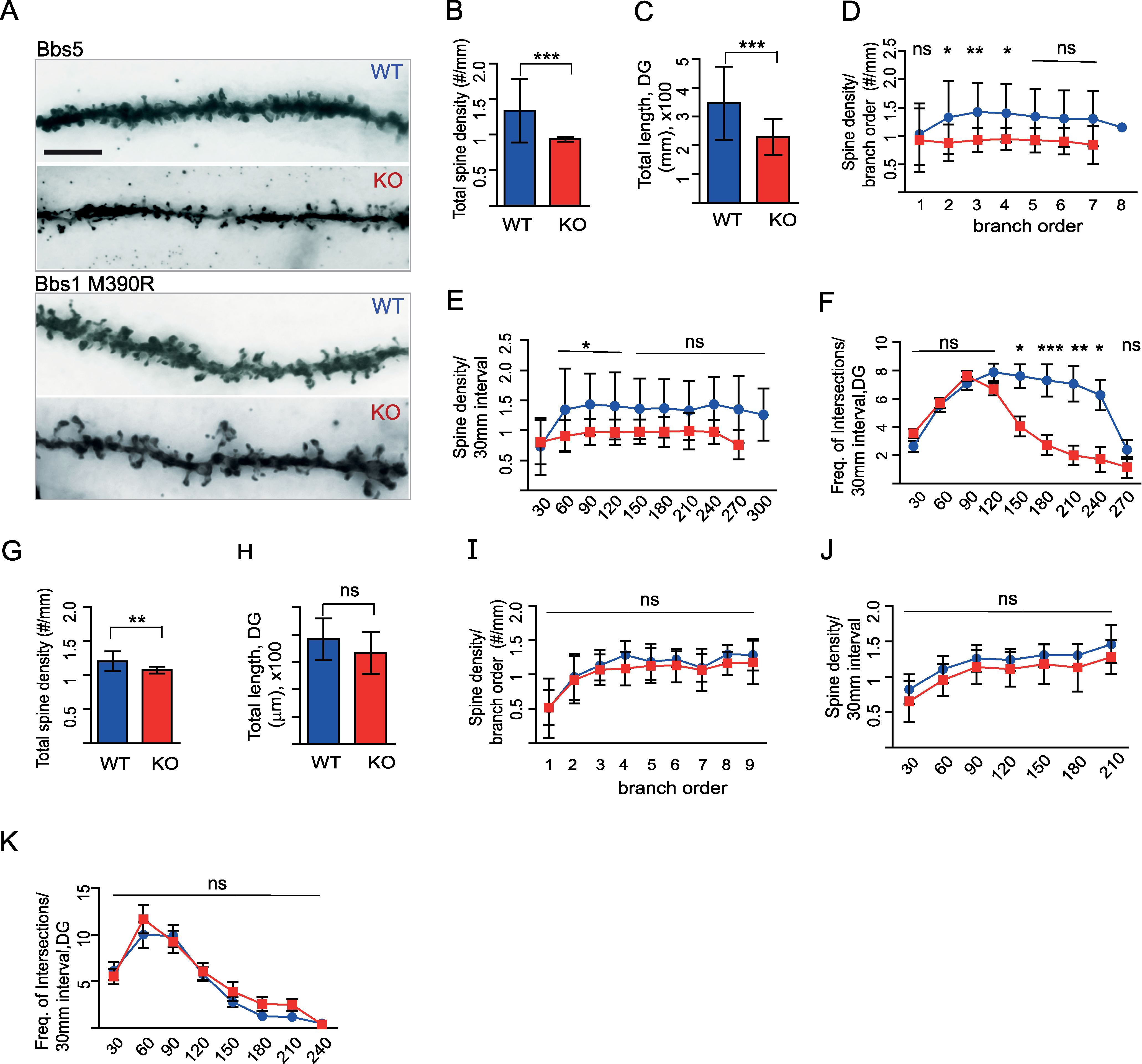

Supplement: S3 Fig — (A) Representative images of Golgi-impregnated DG granule cells of Bbs5 and Bbs1 M390R models. (B-F) Analysis of DG granule cells of Bbs5−/− and Bbs5+/+ mice. (B) Total spine density. (C) Dendritic length. (D) Spine density per branch order. (E) Spine density per 30-μm interval. (f) Frequency of intersections per 30-μm interval. (G-K) Analysis of DG granule cells of Bbs1M390R/M390R and Bbs1+/+ mice. (G) Total spine density. (H) Dendritic length. (I) Spine density per branch order. (J) Spine density per 30-μm interval. (K) Frequency of intersections per 30-μm interval. Biological samples: NWT/Bbs5 = 5; NKO/Bbs5 = 5; total number of analysed cells: NWT/Bbs5 = 22; NKO/Bbs5 = 25; NWT/Bbs1 = 3; NKO/Bbs1 = 3; total number of analysed cells: NWT/Bbs5 = 15; NKO/Bbs5 = 15; mean ± SD, *P < 0.05, ***P < 0.01; one-way ANOVA, Tukey post hoc test. Scale bar, 5 μm. Underlying data are available in S2 Data. Bbs, Bardet-Biedl syndrome; DG, dentate gyrus; KO, knockout; WT, wild type. (TIF) [file pbio.3000414.s005.tif]

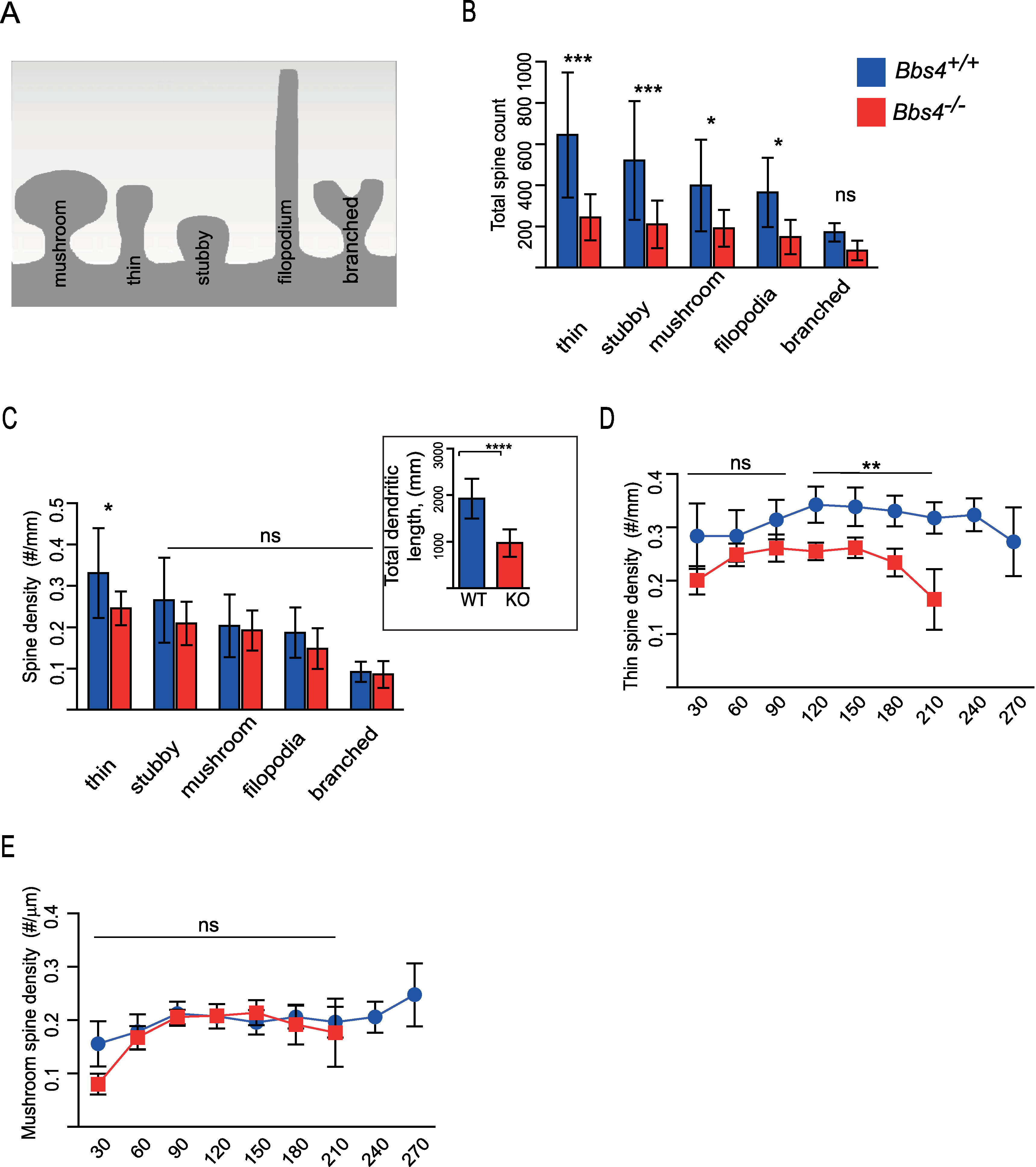

Supplement: S4 Fig — (A) Illustration of dendritic spine subclasses. Adopted from [22]. (B) Total count of ‘thin’, ‘stubby’, ‘mushroom’, ‘filopodia’, and ‘branched’ spines. (C) Total spine density of thin, stubby, mushroom, filopodia, and branched spines. Density is calculated as the number of spines per micrometre of dendrite. Boxed panel shows the reduction in dendritic length. (D) Spine density of ‘thin’ and ‘mushroom’ spines (Nmice/WT = 3; Nmice/KO = 3, Ncells/WT = 15, Ncells/KO = 15, mean ± SD, ***P < 0.001; **P < 0.01; *P < 0.05); one-way ANOVA, Tukey post hoc test. Underlying data are available in S2 Data. Bbs4, Bardet Biedl syndrome 4; DG, dentate gyrus; KO, knockout; WT, wild type. (TIF) [file pbio.3000414.s006.tif]

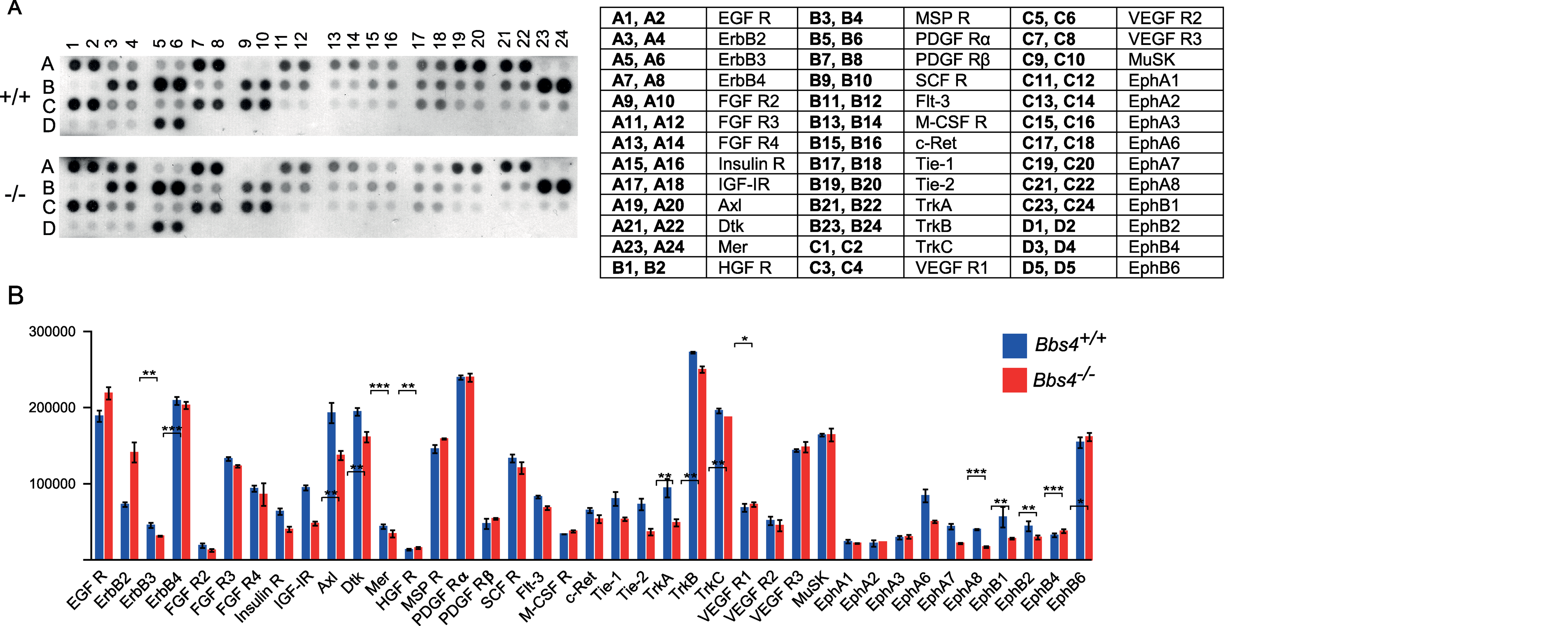

Supplement: S5 Fig — (a) An image of the Phospho-RTK array with annotation box. (b) Quantitative dot-blot analysis reveals significant decrease in phosphorylation of a number of RTKs in P7 of Bbs4−/− synaptosomal fraction (N = 3, mean ± SD); unpaired t test; ImageJ software. Underlying data are available in S2 Data. Bbs4, Bardet Biedl syndrome 4; RTK, tyrosine kinase receptor. (TIF) [file pbio.3000414.s007.tif]

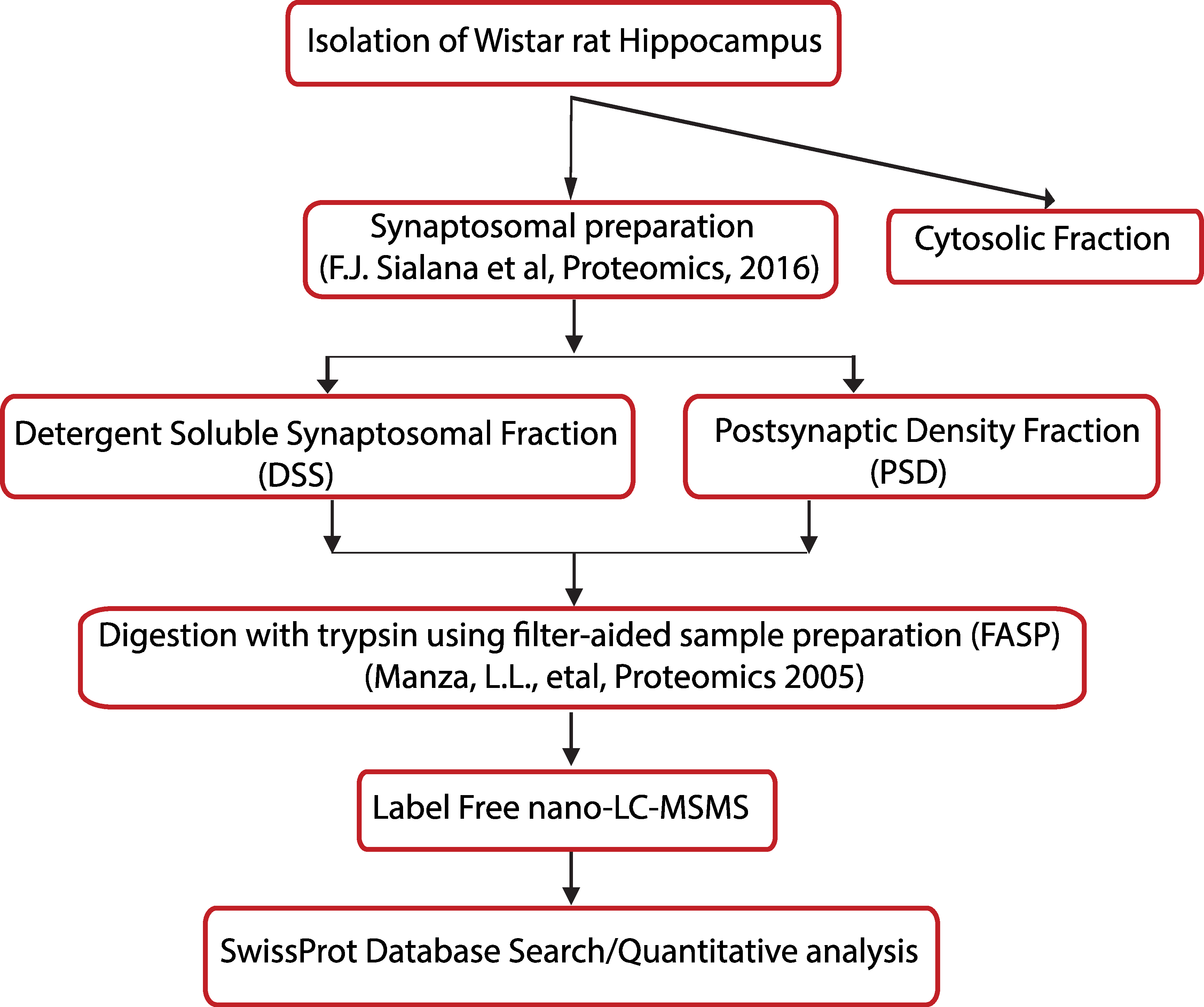

Supplement: S6 Fig — Cytoplasmic and synaptosomal fractions were isolated from the cortices of three Wistar rats. Synaptosomes were further separated into DSS and PSD fractions. A FASP protocol adapted for synaptic membrane proteins is coupled to a gel-free LC-MS to allow analyses of synaptosomal fractions. Database search was performed with search engines against the rat SwissProt protein database. Quantitative information was determined using the software tools, Proteome Discoverer and Isobar. DSS, detergent-soluble synaptosomal; FASP, filter-aided sample preparation; LC-MS, liquid chromatography tandem mass spectrometry; PSD, postsynaptic density. (TIF) [file pbio.3000414.s008.tif]

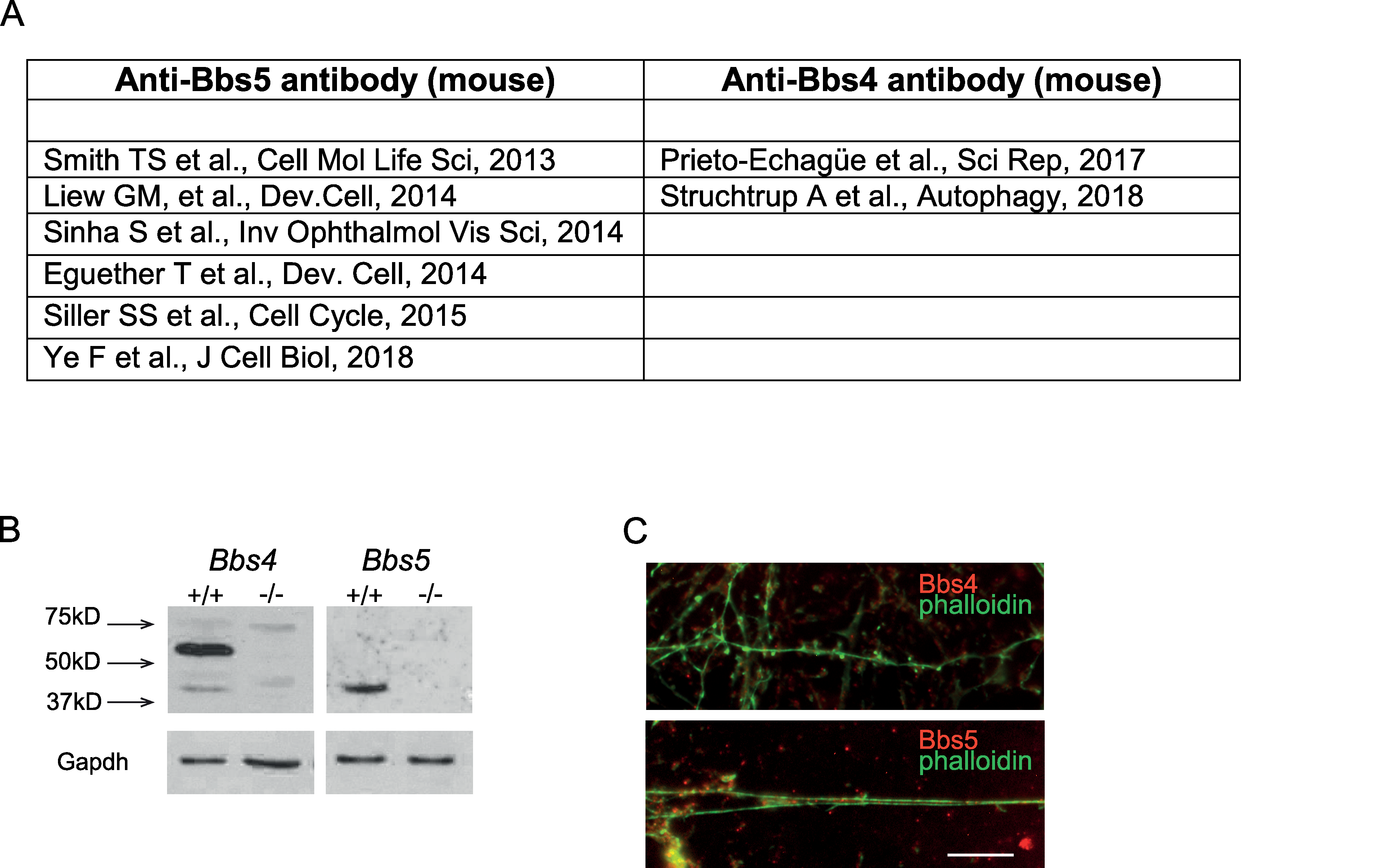

Supplement: S7 Fig — (A) List of published validations of Bbs4 (12766-1-AP) and Bbs5 (14569-1-AP) ProteinTech antibodies used in this study. (B) Total protein extracts of the Bbs4−/−, Bbs4+/+, Bbs5−/−, and Bbs5+/+ mice were immunoblotted with Bbs4 and Bbs5 antibodies as indicated. Approximate molecular weights are listed on the left side. Gapdh was used as the loading control. Wild-type Bbs4 and Bbs5 mice showed a specific single band. Western blot with Bbs4 and Bbs5 antibodies did not detect any specific band in Bbs4 and Bbs5 knockout mice. (C) Images of Bbs4−/− and Bbs5−/− dissociated neurons immunolabelled with anti-Bbs4 and anti-Bbs5 antibodies. Bbs, Bardet-Biedl syndrome; Gapdh, Glyceraldehyde 3-phosphate dehydrogenase. (TIF) [file pbio.3000414.s009.tif]
